# Deep Sequencing and Microarray Hybridization Identify Conserved and Species-Specific MicroRNAs during Somatic Embryogenesis in Hybrid Yellow Poplar

**DOI:** 10.1371/journal.pone.0043451

**Published:** 2012-08-29

**Authors:** Tingting Li, Jinhui Chen, Shuai Qiu, Yanjuan Zhang, Pengkai Wang, Liwei Yang, Ye Lu, Jisen Shi

**Affiliations:** The Key Laboratory of Forest Genetics and Gene Engineering of the Ministry of Education, Nanjing Forestry University, Nanjing, China; East Carolina University, United States of America

## Abstract

**Background:**

To date, several studies have indicated a major role for microRNAs (miRNAs) in regulating plant development, but miRNA-mediated regulation of the developing somatic embryo is poorly understood, especially during early stages of somatic embryogenesis in hardwood plants. In this study, Solexa sequencing and miRNA microfluidic chips were used to discover conserved and species-specific miRNAs during somatic embryogenesis of hybrid yellow poplar (*Liriodendron tulipifera*×*L. chinense*).

**Methodology/Principal Findings:**

A total of 17,214,153 reads representing 7,421,623 distinct sequences were obtained from a short RNA library generated from small RNAs extracted from all stages of somatic embryos. Through a combination of deep sequencing and bioinformatic analyses, we discovered 83 sequences with perfect matches to known miRNAs from 33 conserved miRNA families and 273 species-specific candidate miRNAs. MicroRNA microarray results demonstrated that many conserved and species-specific miRNAs were expressed in hybrid yellow poplar embryos. In addition, the microarray also detected another 149 potential miRNAs, belonging to 29 conserved families, which were not discovered by deep sequencing analysis. The biological processes and molecular functions of the targets of these miRNAs were predicted by carrying out BLAST search against *Arabidopsis thaliana* GenBank sequences and then analyzing the results with Gene Ontology.

**Conclusions:**

Solexa sequencing and microarray hybridization were used to discover 232 candidate conserved miRNAs from 61 miRNA families and 273 candidate species-specific miRNAs in hybrid yellow poplar. In these predicted miRNAs, 64 conserved miRNAs and 177 species-specific miRNAs were detected by both sequencing and microarray hybridization. Our results suggest that miRNAs have wide-ranging characteristics and important roles during all stages of somatic embryogenesis in this economically important species.

## Introduction

Plant microRNAs (miRNAs) are 20- to 24-nucleotide (nt) non-coding RNAs that regulate gene expression at transcriptional and post-transcriptional levels, either by endonucleolytic cleavage or by translational inhibition [1]. Plant miRNAs are high-level regulators of gene expression that affect numerous aspects of plant biology, especially developmental patterning [2]. MiRNAs have a substantial impact on plant development [3], [4]. Increasing evidence indicates that miRNAs play important roles in plant embryo development. During embryo development in loblolly pine (*Pinus taeda*), several miRNAs and their potential mRNA targets showed very different expression patterns in zygotic embryos and female gametophytes [5]. In somatic embryogenesis of *Citrus*, ten conserved miRNAs showed stage- and tissue-specific expression in different embryonic tissues [6]. In *Arabidopsis*, miRNAs enable proper embryonic patterning by preventing precocious expression of differentiation- promoting transcription factors [7]. One or more miRNA targets may sit at the top of the regulatory cascade controlling both activators and repressors of embryonic maturation, suggesting that miRNAs are key regulators of the timing of the maturation program in embryogenesis [8]. Most previous research on miRNAs during embryogenesis has focused on several conserved miRNAs or was based on the model plant *Arabidopsis*. A general view of miRNA varieties that participate in embryogenesis and knowledge of their regulation patterns are much needed. Hard wood plants have a more complex hereditary background and regulation mechanisms, but little is known about miRNA-mediated modulation in early-stage embryonic tissues in these plants. For economically important species, understanding the mechanism of miRNA regulation in embryogenesis would have a positive impact on hardwood plant production.

**Figure 1:**
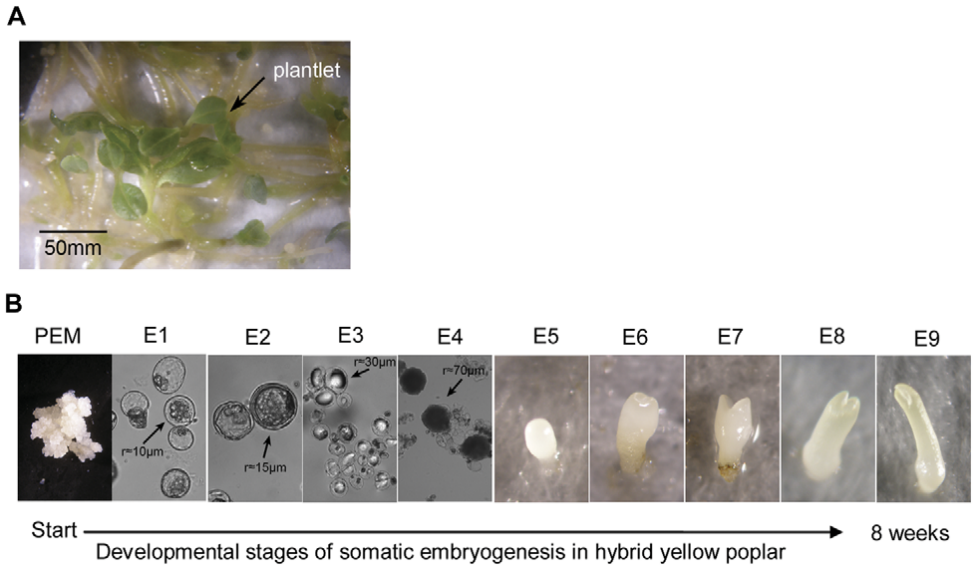
Hybrid yellow poplar (*L. tulipifera*×*L. chinense*) plantlets and somatic embryos. Plantlets generated by somatic embryogenesis (A), and somatic embryos at sequential developmental stages (B). PEM, pre-embryonic mass; E, embryo stage.

Hybrid yellow poplar (derived from the sexual hybridization between *Liriodendron tulipifera* and *Liriodendron chinense*) is a fast-growing hardwood tree that is desirable for the rapid production of forest products, biomass for energy, and phytoremediation purposes [9]–[11]. It belongs to the order *Magnoliales*, with a “basal angiosperm” position in plant phylogeny that makes it an ideal candidate for comparative studies of the evolutionary history of flowering plants [11]–[14]. Since hybrid yellow poplar was regenerated via somatic embryogenesis in 1993 [15], the culture methods have gradually improved [10]. Somatic embryogenesis is a tool used in the study of plant embryology, as it is possible to manipulate cultured cells of many plant species to produce somatic embryos (SEs) in a process that is remarkably similar to zygotic embryogenesis [16]. We have established a high-frequency, precisely controlled somatic embryogenesis system capable of providing synchronized embryos at specific developmental stages (**Figure 1**). This system enables the affordable study of molecular mechanisms during early embryonic development in hardwood species, in contrast to the difficulties in studying zygotic embryos.

The *Liriodendron* genome is not completely sequenced, and the number of expressed sequence tags (ESTs) in the database is limited. Only a few miRNA species in *Liriodendron* have been reported in flowers, leaves, and xylem tissues [14], [17]. Computational prediction is not an effective method for discovering miRNAs that exist during somatic embryogenesis in hybrid yellow poplar. Next-generation high-throughput sequencing enables the exploration of small RNA (sRNA) populations [18]. Many laboratories adopted this technology to study sRNAs in economically important species that lack adequate genome information, such as Chinese yew (*Taxus chinensis*) [19], longan tree (*Dimocarpus longan*), and hybrid yellow poplar(*Liriodendron tulipifera*×*L. chinense*). Microarray analysis, another high-throughput technology, can detect and confirm the quantity of miRNAs with known sequences. In this study, probes designed from the Solexa sequencing results were used for subsequent microarray analyses to identify miRNAs during somatic embryogenesis in hybrid yellow poplar. The potential biological processes and molecular functions of the miRNA targets were predicted by BLAST analyses with *A. thaliana* GenBank sequences and subsequently analyzed with Gene Ontology. An overview of the miRNA varieties and their potential functions during the early developmental stages of somatic embryogenesis in hybrid yellow poplar is presented.

## Results and Discussion

### Complex sRNA population detection in hybrid yellow poplar SEs

To date, little genomic research has been conducted in *Liriodendron*. Only 24,132 ESTs, mainly from floral tissues of *L. tulipifera*, have been deposited in the National Center for Biotechnology Information EST database [20], [21]. Few ESTs of *L. chinense* or *L. tulipifera*×*L.chinense* are publicly available. The limited number of ESTs made it difficult to perform a comprehensive study of hybrid yellow poplar miRNAs using only a computational analysis, and hence Solexa sequencing technology was used to directly obtain information on sRNAs.

Sequencing of sRNA pools collected from each stage of somatic embryogenesis yielded a total of 17,214,153 raw reads and consisted of 7,421,623 unique sequences. After removing the adapter and low-quality sequences, 10,348,953 sequences were obtained with lengths ranging from 15 to 26 nucleotides. Of these sequences, 1,679,075 were aligned to rRNA, tRNA, sno/snRNA, mRNA, other non-coding RNAs, and repeat regions. After further removing the above sequences, the remaining 8,669,878 reads were used for miRNA identification. Although some small RNAs were very high in abundance and presented thousands of times in our dataset, a large proportion of small RNAs were sequenced only a few times. For example, 4,825,663 out of 15,174,616 small RNAs were sequenced less than 3 times in our experiment. The results show that the expression of different sRNAs in hybrid yellow poplar varies drastically and suggest that the somatic embryo tissues of hybrid yellow poplar contain a large and diverse sRNA population.

**Figure 2:**
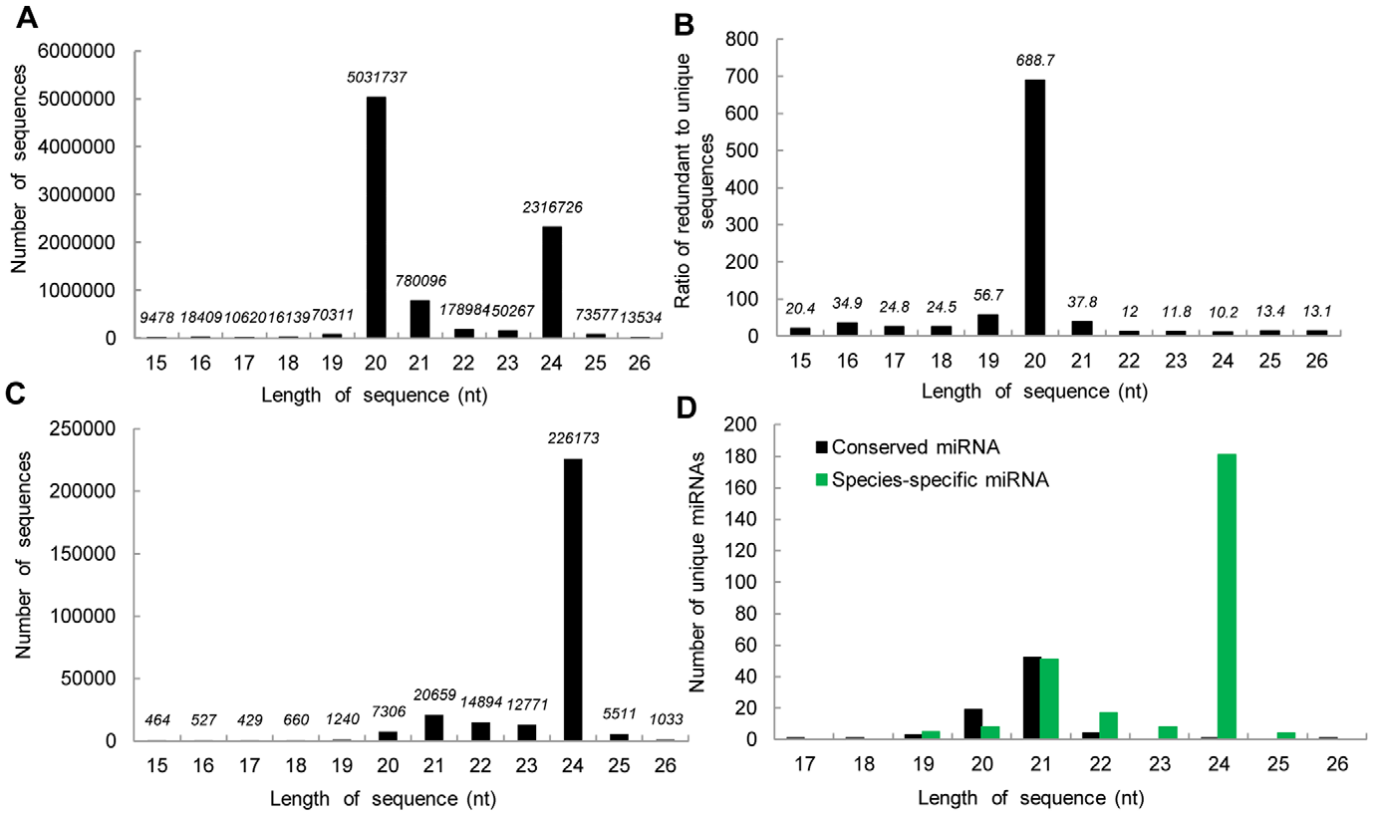
Sequence length distribution in hybrid yellow poplar. Length distribution and abundance of redundant (A) and unique (C) sequences; (B) the ratio of redundant to unique sequences at each length; (D) Length distribution of the conserved and species-specific miRNAs.

The size distribution analysis of all sRNAs is summarized in Figure 2. Most of the raw sequences were distributed between 19 and 24 nt, with 20 nt (58.0%) and 24 nt (26.7%) being the predominant size classes (**Figure 2A**). Because these reads included a large number of redundant sequences, the ratio of mappable raw and unique sequences was calculated. Most of these sequences were redundant, with 20-nt sequences reaching 688.7-fold redundancy, a higher value than for any other sequence length (**Figure 2B**). The 20-nt sequences may have had such high redundancy because of the ancient hereditary background, or this may be a bias induced by uneven sequencing efficiency [22], [23]. The size distribution of unique sequences differed from the redundant ones and varied widely. There were significantly more 24-nt sequences (77.54% of all sequences), followed by 21-nt, 22-nt, 23-nt, and then 20-nt sequences (**Figure 2C**). Similar to *Arabidopsis*, most of the sRNAs that acted as small interfering RNAs (siRNAs) associated with DNA silencing were preferentially 24 nt long [24]. This result was also consistent with those of *Oryza sativa* [25], *Solanaceae* [26], *Medicago truncatula* [27], *Citrus trifoliate* [28], and *Arachis hypogaea L.* [29]. The length distribution was compared between conserved and species-specific miRNAs (**Figure 2D**), both of which had 51 or 52 unique sequences for the typical 21-nt plant miRNA. Most of the 24-nt unique sequences were species-specific miRNAs. This result suggested that most miRNAs discovered in hybrid yellow poplar corresponded to non-conserved, previously unidentified miRNAs.

### Conserved miRNAs in hybrid yellow poplar SEs

**Figure 3:**
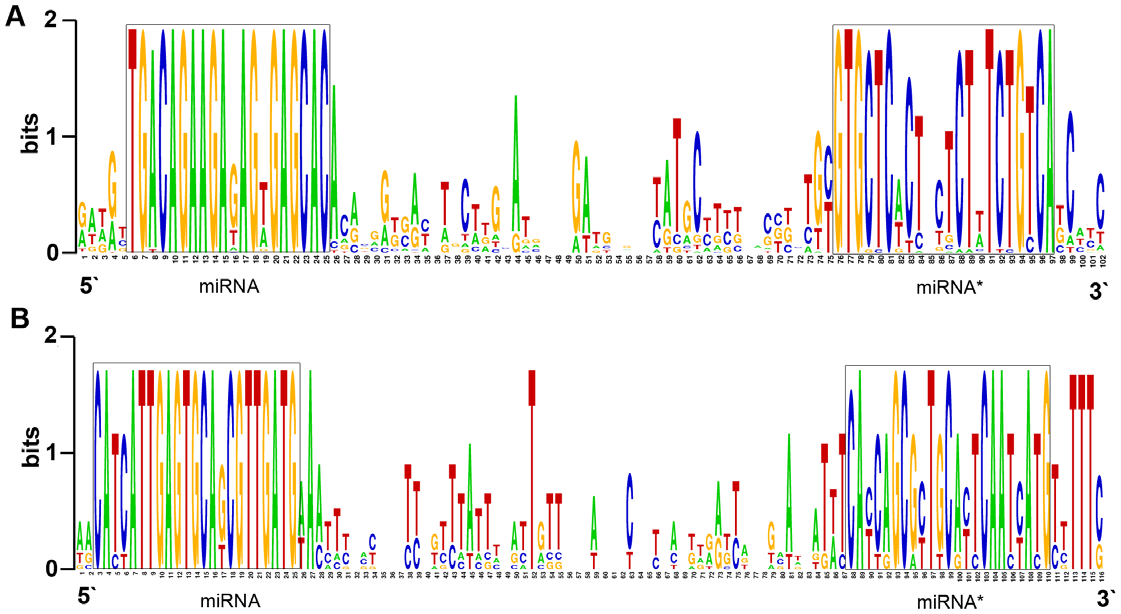
Conservation of hybrid yellow poplar miRNAs. The miRNA conservation was studied by aligning of pre-miRNAs of *P. trichocarpa*, *Arabidopsis*, *Oryza*, *V. vinifera* and *liriodendron*, using Weblogo software. (A) miRNA156; (B) miRNA397. The mature sequences are highlighted in a rectangle box.

The miRNA database miRBase (release 17, April 2011) contains 3,362 miRNAs from 46 plant species. Numerous miRNAs have been identified from the sequenced genomes of *A. thaliana* (232), *O. sativa* (491), *M. truncatula* (375), *Populus trichocarpa*(234), and *Vitis vinifera* (163). The genome of the hybrid yellow poplar has, however, not been fully sequenced. Hence, in this study, all sRNA sequences were BLASTn searched against the currently known miRNAs in miRBase, using 0–3 mismatched bases in the mature region. All of these miRNAs were expended for appropriate length on the plant genomes or ESTs and supported with satisfactory secondary structures. Plant miRNAs are highly conserved among different plant species, both in primary and mature miRNA forms, especially for mature sequences and their complementary miRNA* sequences [30]. As miR156 and miR397 detected in our study, they showed conservation with *Arabidopsis*, *P. trichocarpa*, *O. sativa* and *V. vinifera* (**Figure 3**). The conserved nature of miRNAs made it a logical approach to classify new miRNAs by comparing their precursors and mature regions to other species [30]–[32]. Therefore the predicted conserved miRNAs were falling into families by using the stem-loop precursors aligning to the RNA family database Rfam (http://www.sanger.ac.uk/Software/Rfam/) [33]–[35] and miRBase, then the BLAST results were reconfirmed by making a multiple sequence alignment (MSA) (http://weblogo.berkeley.edu/logo.cgi) [36] analysis. After BLAST searches and sequence analysis, a total of 83 conserved miRNAs belonging to 33 miRNA families that are conserved across a variety of plant species were identified in hybrid yellow poplar. Most of these miRNAs were 21 nt long and started with a “U” at their 5′ end (**Table S1**). 22 miRNAs (including 12 conserved and 10 species-specific miRNAs) were checked by using stem-loop reverse transcription–polymerase chain reaction(RT-PCR) [37], clones containing the mature regions of these miRNAs were obtained (**Figure S1**) and 4 out of 10 species-specific miRNAs only got partial sequences of the mature region(≤12), however, the results from RT-PCR verified the existence of these predicted miRNAs in hybrid yellow poplar [38]. Some of these miRNAs yielded inconsistent results in current miRBase when they were searched by their pre-miRNA and mature miRNA sequences respectively, for serious consideration, we didn't show them in our supplementary file. Comparative analysis showed that 20 miRNA families in our data were conserved in *Arabidopsis*, *V. vinifera*, *P. trichocarpa*, and *O.sativa*. In contrast, 20 sequences were shared among three or fewer plant species. In addition, miR894, miR1510, miR1511, and miR2911 were not found in these four species but were conserved with miRNAs from *Physcomitrella patens*, *Glycine max* and *Populus euphratica* respectively.

**Figure 4:**
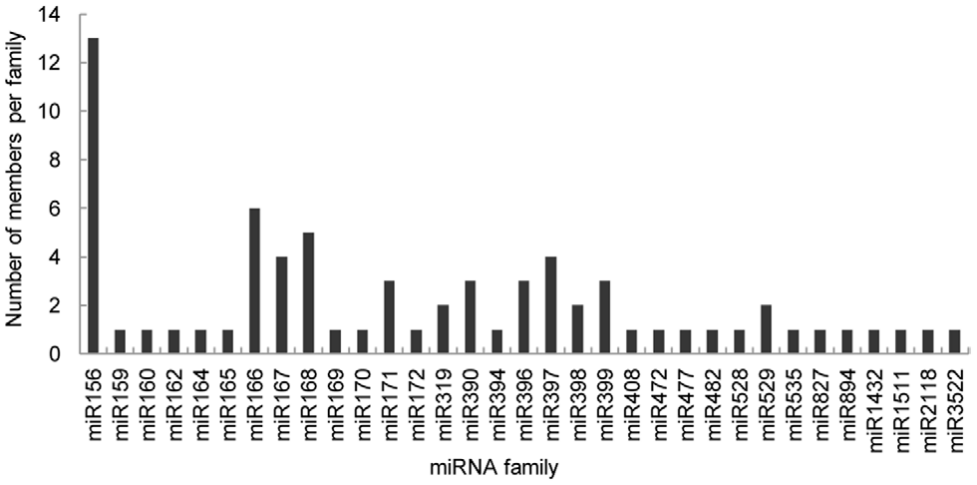
Number of identical miRNA members in each family in hybrid yellow poplar.

Analysis of millions of hybrid yellow poplar sRNA sequences enabled the identification of various miRNA families and different members within a given family. Most of these homologous miRNAs had some sequence differences. Highly expressed miRNAs would be likely to have a large number of sequenced clones. The 33 conserved miRNA families consisted of 1–13 different members (**Figure 4**). Among these families, the miR156 family had the most family members (13), with 93.7% of all conserved miRNA reads, followed by miR166 (6), miR168 (5), miR167 (4), miR397 (4), miR390 (3), and miR399 (3) etc. The remaining miRNA families had only one or two members. The reads of different members within a given family were not evenly distributed: miR156 with 13 family members had 47 to 4,750,907 reads; miR166 with 6 members had 10 to 216,840 reads; miR390 with 3 members had 5 to 2,768 reads. These results indicate that there was potential functional divergence within families, whereby one or several members within a family would be the main products in the pre-miRNA cutting process and would have the major role. In our study, the sequencing material consisted of total RNA pools collected from each stage of somatic embryogenesis, the detailed expression of a given miRNA at a certain developmental stage could not be identified, but some miRNA families exhibited dominant place in family members and copy reads, they presumably played an important role in this process, such as miR156 and miR166. Our speculation was in accordance with miRNA research in *Arabidopsis* which showing that miR156 is active throughout early embryogenesis, and it is a key medium in the repression of precocious gene expression and pattern formation during embryogenesis [7]. miR166-mediated regulation of *PHB* and *PHV* is also important during early embryonic patterning [33]. The similar results are understandable because we used mostly early-stage SE tissues and morphogenesis-to-maturation-phase transitions occur frequently in somatic embryogenesis.

**Figure 5:**
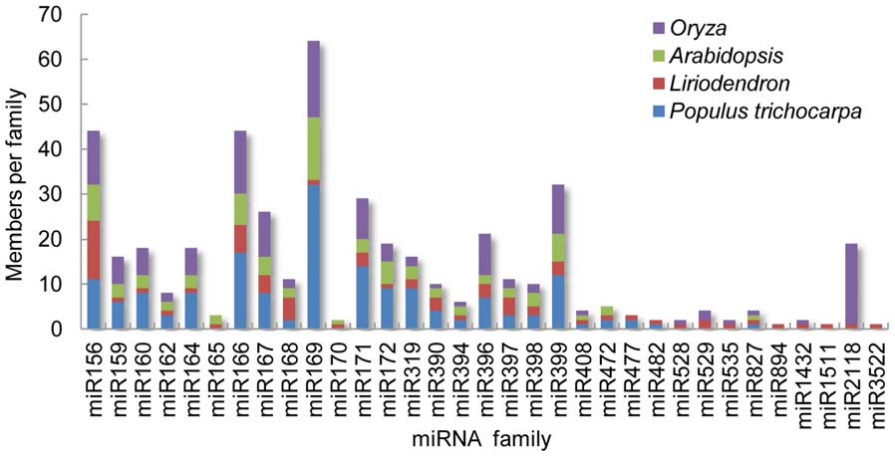
Paralogs per conserved miRNA family in hybrid yellow poplar, *Arabidopsis*, *P.trichocarpa*, and *Oryza*. The number of family members in *Arabidopsis*, *P. trichocarpa*, and *Oryza* miRBase and the number of family members in hybrid yellow poplar from deep-sequencing results in this study are shown.

We compared the number of miRNA family members isolated from deep sequencing with those from *Arabidopsis*, *P. trichocarpa*, and *O. sativa* miRNAs available in miRBase (**Figure 5**). Based on this comparison, we believed that many miRNAs had not yet been discovered in hybrid yellow poplar. A miRNA microarray analysis would prove this presumption.

### Identification of species-specific miRNAs in hybrid yellow poplar SEs

**Figure 6:**
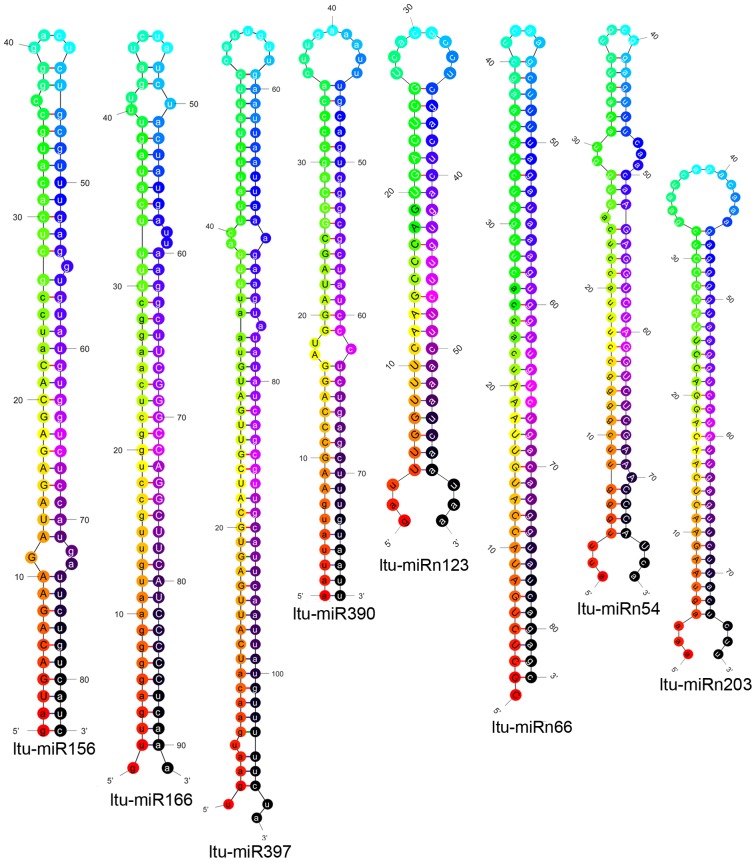
Predicted secondary structures of identified miRNAs in hybrid yellow poplar. The uppercase letters refer to mature sequences. The secondary structures are generated from the *Liriodendron* genome and EST sequences.

In addition to the identification of conserved miRNAs, 273 species-specific miRNAs which mapped only to the *liriodendron* genome were also identified (**Table S2**). Plant miRNA precursors had lower similarity and were less conserved than other RNAs although mature miRNAs are highly conserved, but all miRNA precursor sequences have a typical stem-loop structure [39], [40] and lower folding free energies than do random sequences of other non-coding RNAs of a certain length [41]. Besides, miRNA precursors and mature miRNAs contain more “A+U” nucleotides than “G+C” nucleotides to facilitate processing into mature miRNAs by the RNA-induced silencing complex (RISC) and to serve as a signal for miRNA biogenesis [42]. In our study, all sequences were mapped to the *Liriodendron* genome and ESTs with no more than two mismatches. Their precursors (pre-miRNAs) were predicted by RNAfold software (http://mfold.rna.albany.edu/?q=mfold/ RNA-Folding-Form) using <300 bp RNA (16–260 bp). Similar to conserved miRNAs, the sequences had significant stem-loop hairpin secondary structures, the structures of several predicted miRNA precursors were showed in Figure 6. The folding free energy of these hairpin structures ranged from −9.5 to −123.1 kcalmol^−1^ (average of −38.8 kcalmol^−1^) with an average 44.3% “G+C” content, and 76% of these sequences had a higher “A+U” content than “G+C” content. In addition, the minimal folding free energy index (MFEI; −dG×100/mirLen/CG%) value was used to distinguish miRNA from other types of coding or non-coding RNAs. For most miRNA precursors, the MFEI is >0.85 [43], [44]. In our study, the MFEI was 0.4 to 1.7, with most MFEIs>0.85.

The length distribution of these species-specific miRNAs varied from 19 to 25 nt, with 24 nt (66%) and 21 nt (18.5%) as the major size classes, followed by 22 nt (6.5%) and the remaining size classes with only ∼9% overall (**Figure 2D**). This two-peak distribution (21 and 24 nt) is quite common for plant sRNA sequencing results [27]–[29], [45], [46]. Although this study identified more species-specific miRNAs (273) than conserved miRNAs (83), the read number for each species-specific miRNA (3 to 10,109) was much lower than that for the conserved miRNAs (3 to 4,750,907). Of the species-specific miRNAs, 80% had <10 reads and 35% had only 3 reads, whereas nearly 81% of conserved miRNAs had >10 reads and 46.6% of those miRNAs had >100 reads. The expression patterns of most non-conserved miRNAs in hybrid yellow poplar may be related to tissue or developmental-stage specificity. Further studies on their target biological functions may reveal if these species-specific miRNAs are key regulators in somatic embryogenesis in hybrid yellow poplar as compared with other developmental processes or plant species.

### Validation and complementarity of potential miRNAs by microarray analysis

**Figure 7:**
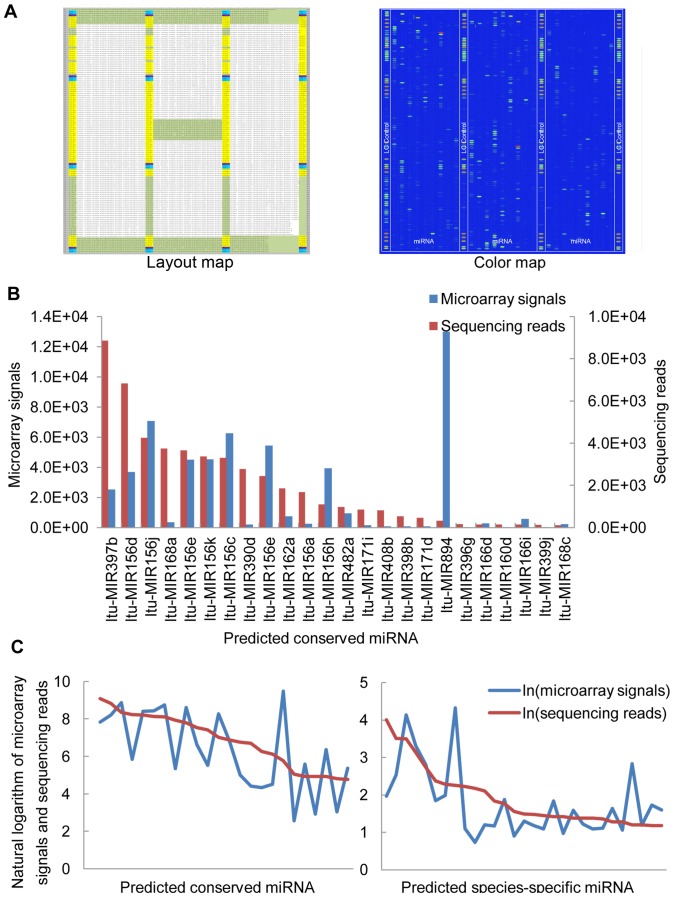
Relationship between the miRNA microarray signals and deep-sequencing reads. (A) Layout diagram (left) and color map (right) of the miRNA microarray. The empty control, spike-calibration control and biosequence control are colored in purple, bright blue and yellow respectively. The probes ordered according to the sequencing result are colored in green. (B) Comparison of microarray signals (blue) and sequencing reads (red) for the same predicted conserved miRNAs. (C) Analysis of the relationship between microarray signals and sequencing reads under natural logarithm in 24 conserved miRNAs (left; miRNAs are ordered as in B) and 26 species-specific miRNAs (right).

MicroRNA microarray analysis is a high-throughput technology for detecting and confirming the quantity of miRNAs for which sequence information is known. In our study, the deep-sequencing strategy generated hundreds of predicted miRNA sequences. To validate the sequencing results and obtain a more comprehensive and high-throughput analysis of miRNAs expressed during somatic embryogenesis in hybrid yellow poplar, a mixed RNA pool microarray was used, which consisted of 1,749 probes including 299 deep sequencing–predicted miRNA sequences and 1,450 unique, mature plant miRNA sequences (based on Sanger miRBase release 15.0) (**Figure 7A**). The results of the microRNA microarray analysis are as follows. (1) 1,024 sequences (58.5% of the ordered sequences) were detected with a coefficient of variation less than 0.5 and a signal-to-noise value more than 4.59. Among these detectable transcripts, 64 predicted conserved miRNAs and 177 predicted species-specific miRNAs were detected by both sequencing and microarray hybridization. In the 64 predicted conserved miRNAs, 20 miRNA sequences including 9 miRNA families' (miR894, miR156, miR159, miR2118, miR397, miR1511, miR535, miR529 and miR396) average signal intensity were higher than 1000. Some of these high-expressed sequences were also verified by stem-loop RT-PCR (**Figure S1**). (2) Using the mean value of background signal intensities plus two standard deviations as the threshold (38.53) [47], [48], 149 unique sequences were considered to be new potential conserved miRNAs detected by the microarray only (**Table S3**). Compared with the sequencing results, each miRNA family was expanded. Furthermore, another 29 miRNA families were detected in hybrid yellow poplar. Hence, a total of 232 conserved miRNAs and 273 species-specific miRNAs were detected during somatic embryogenesis in hybrid yellow poplar by either deep-sequencing and/or miRNA microarray hybridization technology.

In our study, we have used both deep-sequencing and microarray technologies with the same total RNA pools to detect and validate sRNAs expressed in somatic embryogenesis. The expression trend of miRNAs detected by these two technologies was different, and the relationship was not quite linear (**Figure 7B, C**). Both of these technologies have advantages and disadvantages [23]. Solexa deep sequencing can obtain sequence information directly and is more sensitive in detecting sRNAs with low copy numbers. But the depth of sequencing required to sample the transcriptome effectively, and the two PCR steps in the sequencing process (bridge-PCR and sequencing-by-synthesis PCR) would skew some of the middle and low reads farther from the real expression state. The high costs can also be prohibitive for doing biological replicates. In addition, the data processing results in many low-copy sequences being discarded, as in our study, whereby 4,513,438 unique sequences with 60.81% of the total unique sequences were discarded because of their low copy numbers (<3), and this low expression may have been caused by an inefficient ligation or PCR process. Therefore, the sRNA reads detected by deep sequencing have some errors because of stochastic events, and optimum analytical strategies still need to be developed. In contrast, microarray analysis is a direct measure of the expression of sRNAs and has more-mature analysis strategies, but it may be less sensitive. This may be why some miRNAs had different expression trends in these two detection systems and why some miRNAs were detected by microarray analysis but not by deep sequencing. Combining these two technologies can circumvent their individual shortcomings and is an effective method for discovering and validating a relatively large number of miRNAs in plant species that lack significant gene sequence information.

### Putative functions of predicted miRNA targets in hybrid yellow poplar

**Figure 8:**
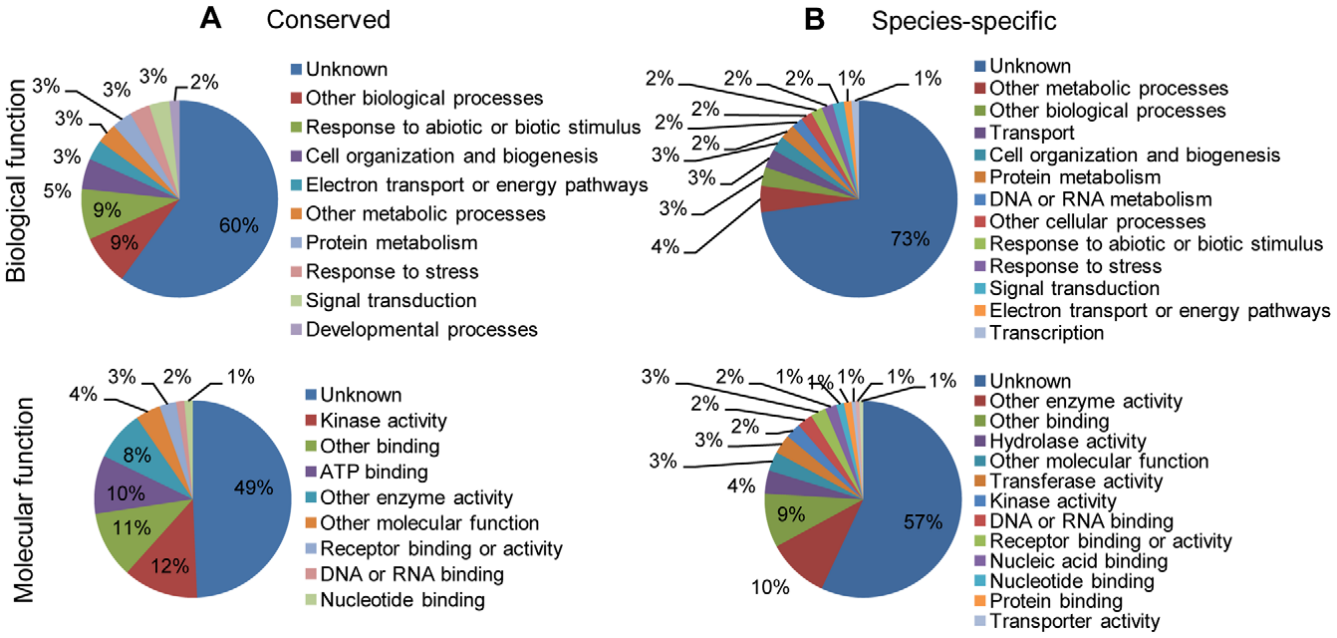
Putative molecular functions and biological processes of predicted conserved and species-specific miRNA targets. The miRNA targets in hybrid yellow poplar were predicted by carrying out BLAST search of *A. thaliana* and *Liriodendron* EST databases with psRNATarget tool and then analyzed using Gene Ontology.

The potential biological processes and molecular functions of the targets of these conserved and species-specific miRNAs were predicted by carrying out BLAST search against *A. thaliana* and *Liriodendron* EST databases and then analyzing the results with Gene Ontology (**Figure 8**). The predicted target genes were involved in many metabolic and biological processes, including cell organization, electron transport and energy pathways, signal transduction, protein metabolism, responses to an abiotic or biotic stimulus, growth, and many other developmental processes. The molecular functions of these target genes included involvement in enzyme activity, ATP binding, DNA or RNA binding, nucleotide binding, receptor binding or activity, and other functions. Most of the predicted miRNA target functions were unknown (**Figure 8**).

Somatic embryogenesis is a process that involves the regulatory roles of many miRNAs [16]. Deeply conserved miRNA families are integral components of functional regulatory network sat all plant development stages [49]. Many miRNAs target transcription factors, which form certain DNA-binding structures that regulate gene expression. For example, miR156 targets the *SQUAMOSA PROMOTER BINDING PROTEIN-LIKE*(*SPL*) family [50]. MiR156 represses the zygotic expression of both *SPL10* and *SPL11* during early embryogenesis in *Arabidopsis*, which are key repression factors of transcription during the transition from the premature to maturation phase [7]. This was indirectly supported by our research. In early-stage SEs, ltu-miR156 had the most family members and sequencing reads, and each family member also had a high level of expression in the microarray hybridization (**Figure 7B**). These miRNAs targeted *SPL* family members (*SPL3*, *SPL5*, *SPL10*, and *SPL11*), and each ltu-miR156 family member was considered likely to be involved in DNA binding (**Table S4**). According to Willmann [8], multiple miR156-targeted *SPL* genes may redundantly regulate the same targets. Highly conserved miRNA sequences can target different genes between diverse species such as *Arabidopsis* and *P. trichocarpa* [51]. Based on *A. thaliana* annotation, miRNA target genes were found for several conserved miRNAs in hybrid yellow poplar (**Table S4**): *ARF10* (miR160), *CYP96A1* (miR162), *NAC* (miR164), *PHB* and DNA-binding factor (miR165/166), *NF-YA8* (miR169), SCARECROW transcription factor family protein (miR170/171), *SNZ* (miR172), *MYB* (miR319), *GRF* (miR396), copper ion binding (miR408), *SPL11* (miR529) etc. These targets are all important transcription factors that are active in cell differentiation and embryo development.

**Figure 9:**
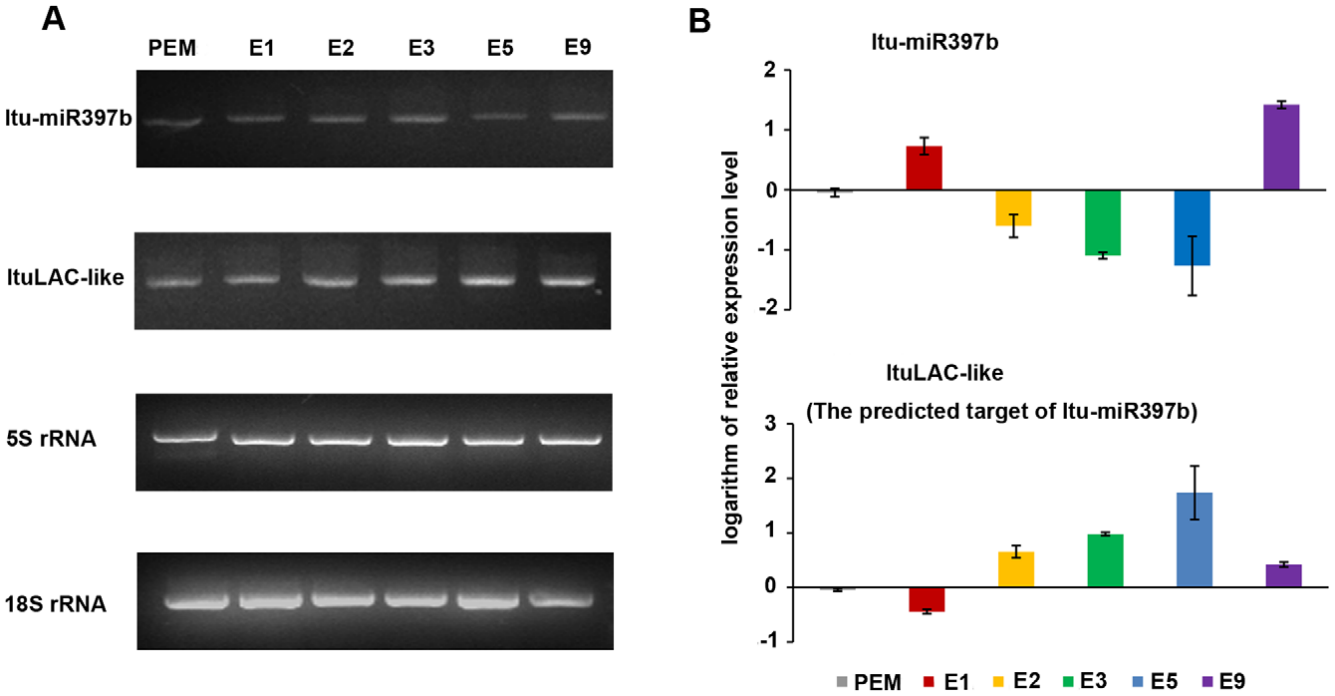
qRT-PCR expression analysis of ltu-miR397b and its predicted target gene in hybrid yellow poplar. (A) ltu-miR397 and the middle region of ltuLAC-like gene were detected in PEM, E1, E2, E3, E5 and E9 tissues by qRT-PCR. Both 5S rRNA and 18S rRNA were used as internal controls. The product of each sample was separated on a 2% (w/v) agarose gel. (B) Relative expression level of ltu-miR397b and ltuLAC-like gene in SEs. Error bars indicate one standard deviation of three different biological replicates (n = 3). All relative quantitative values were plotted on a logarithmic scale.

In our study, for the gene description in *liriodendron* database is limited, only the target of ltu-miR397 (laccase gene) was found annotated when we submitted the small RNA sequences to psRNATarget by setting the *liriodendron* genome as the target searching library. The middle region of laccase gene (*LAC*) included the target site was cloned. Then a quantitative RT-PCR examination between miR397 and the predicted target region was performed. Both 5S rRNA and 18S rRNA were used as the reference genes. The logarithmic processing results showed that the expression level of miR397 became lower from stage E1 to E5 while in *LAC* the opposite was the case (**Figure 9**). Some studies on poplar indicated that laccase is essential for normal cell wall structure and integrity in xylem fibers, and *lac3* gene suppression lead to cell wall detachment in the primary wall and middle lamella region [52]. The experiment on *fucus* proved that cell wall would maintain the differentiated state and to direct cell fate in plant development [53], [54]. In our study, from E1 to E5 is a process that single embryo cell developed into multicellular orgnanism. Thus we speculated that laccase gene family would also play a very important role in the early morphological formation of somatic embryogenesis and miR397 would be one of the key regulators during this process.

In contrast to conserved miRNAs, most candidate species-specific miRNAs were 24 nt with a 5′ adenosine (**Table S5**), similar to the 24-nt siRNAs that guide the direct methylation of target loci DNA in *A. thaliana* [49], [55]–[57]. Furthermore, our study found that ltu-miRn213 and ltu-miRn235 targeted O-methyltransferases, whereas ltu-miRn108, ltu-miRn118, and ltu-miRn222 targeted GTPase activators and GTP-binding proteins. Studies in *P. patens* also link miRNAs to transcriptional gene silencing through DNA methylation [58]. Most miRNA families are lineage restricted or species specific, and the evolution of miRNA genes will influence the evolution of regulatory networks [49]. *Liriodendron* is a primitive category of angiosperm, and hence these species-specific miRNAs may play an important role both in transcriptional silencing pathways and the traditional post-transcriptional pathway.

Combining Solexa deep sequencing with microarray hybridization, we discovered 232 predicted conserved miRNAs and 273 candidate species-specific miRNAs from hybrid yellow poplar SEs. The potential miRNA target functions were related to various biological and metabolic processes. These results indicate that miRNAs play wide-ranging and important roles during all stages of somatic embryogenesis in hybrid yellow poplar. Our study have provided the information of hybrid yellow poplar miRNAs, however, more studies need to be performed to elucidate the functions that these predicted novel miRNAs have in the SEs development.

## Materials and Methods

### Plant materials

The pre-embryonic mass (PEM) and different stages of SEs (**Figure 1B**) were collected sequentially from an improved hybrid yellow poplar (*L. tulipifera*×*L. chinense*) somatic embryogenesis system established by our lab [59]: PEM were induced by immature embryos got from control-pollinated cores (hybrid between *Liriodendron chinense* and *Liriodendron tulipifera*) and maintained on solid induced I medium. Suspension culture embryonic cells and tissues (E1 to E4) were selected by synchronized methods and collected at their transformation stages (about every three days).Then these pre-embryos were germinated on solid induced II medium in a light-controlled growth room and another five stages of embryos (E5 to E9) were harvested weekly. All the callus and embryo tissues were staged using a microscope, then immediately frozen in liquid nitrogen, and stored at −80°C until used.

### RNA isolation and purification

Total RNA was isolated and purified from each stage of somatic embryogenesis using the Total RNA Purification kit (Norgen Biotek Corporation, Canada), according to the manufacturer's instructions and using the on-column DNA removal protocol. All RNA samples from tissues of different SE stages were stored at −80°C until sRNA sequencing was performed and then were mixed in an equal fraction ratio to form a single RNA pool.

### sRNA sample preparation and sequencing

sRNA samples were sequenced by LC Sciences(Hangzhou, Zhejiang, China) using the high-throughput sequencing technology developed by Illumina. The quality of total RNA samples was checked by using the Agilent Technologies 2100 Bioanalyzer. The sRNAs (18–30 nt) were separated by size fractionation on a 15% (w/v) Tris-borate-EDTA urea polyacrylamide gel from 10 µg total RNA. After recovering the isolated RNAs, the purified sRNAs were then ligated to 5′(GUUCAGAGUU CUACAGUCCGACGAUC) and 3′(P-UCGUAUGCCGUCUUCUGCUUG-UidT) chimeric oligonucleotide adapters and reverse transcribed to single-stranded cDNAs with an RT primer (CAAGCAGAAGACGGCATACGA). Subsequently, the products were amplified by PCR. Finally, after purifying and validating the sRNA cDNA library, Solexa sequencing technology was employed to sequence these prepared samples.

### Identification of conserved and species-specific miRNAs

The raw sequences were processed using the Illumina pipeline program. After masking of adaptor sequences and removal of contaminated reads, the clean reads were filtered for miRNA prediction with the ACGT101-miR-v3.5 software package (LC Sciences, Houston, USA) and subsequently analyzed according to http://www.lc-bio.com/products/available_arrays.asp?id=181. Secondary structure prediction of individual miRNAs was performed by MFOLD software (Version 2.38;http://mfold.rna.albany.edu/?q=mfold/RNA-Folding-Form) using the default folding conditions. A small RNA was considered as a potential miRNA candidate only if it met the following criteria [43], [60], [61]:(1) the EST sequence can fold into an appropriate stem-loop hairpin secondary structure and the hairpin length is of ≧50; (2)the small RNA sat in one arm of the stem-loop;(3) the percentage of small RNA in stem region is of ≧80% and the number of basepair in stem region is of ≧16;(4)no more than 2 biased bulges in mature region and the number of allowed biased errors in one bulge in mature region is less than 4;(5)the number of basepair in mature or mature* region is of ≧12;(6) predicted secondary structure had higher minimal folding free energy index and lower minimal folding free energy(≤−15 kcalmol^−1^).

### Microarray hybridization and RT-PCR

Both microarray hybridization and the deep sequencing used the same RNA sample. The miRNA microarray was synthesized *in situ* by LC Sciences (http://www.lcsciences.com/mirna.html), where chip hybridization was also performed. The custom _μ_paraflo™ microfluidic chip contained 1,749 unique plant miRNAs, representing 1,888 miRNAs from 37 plant species listed in Sanger miRBase release15.0(http://www.mirbase.org/) and 299 predicted miRNA sequences detected from our sRNA sequencing results. Each probe was spotted in duplicate in the slide, the control probes that were used for quality controls of chip production, sample labeling and assay conditions were also included in the chip (**Figure 7A**). The hybridization image was digitized using the Array-Pro image analysis software (Media Cybernetics). The data were analyzed by subtracting the background, and the signals were normalized based on the LOWESS program [62]. A transcript to be listed as detectable should meet the following criteria: signal intensity higher than 3×(background standard deviation) and spot coefficient of variation (CV) less than 0.5. CV was calculated by (standard deviation)/(signal intensity). During data process, “bad spots” that have signal values deviated more than 50% of average values of repeating spots and/or spot CV larger than 0.5 were discarded.

RT-PCR was performed using total RNAs from SEs as previously described in Chen [37] and Gasic's [63] stem-loop RT-PCR strategy. The PCR products were detected by gel electrophoresis and then recovered with a small DNA recovery kit (DP4001; Bioteke). Subsequently, the purified PCR products were cloned into pMD 19-T Vector (D102A; Takara) and sequenced by Invitrogen. Quantitative real time PCR was performed using the Power SYBR Green PCR Master Mix kit (Applied Biosystems, USA) on an ABI 7500 Real-Time PCR System (Applied Biosystems, USA). RQ (Relative Quantitation) gene expression analysis was performed by Sequence Detection Software. Both 5S rRNA and 18S rRNA were used as internal controls and all reactions were run in triplicate. All the primers used in RT-PCR and qRT-PCR were listed in Table S6.

### Target gene prediction and ontology analysis

All miRNAs from both deep sequencing and microarray analysis were submitted to psRNATarget (http://plantgrn.noble.org/psRNATarget/) using the “*A. thaliana* TAIR9, cDNA, removed miRNA gene, released 06/19/2009” [64], [65] as the sequence library for target search. Maximum expectation (2) and complementarity scoring (17) values were used. A statistical analysis showing the probability of target enrichments in some biological processes and molecular functions was conducted using Gene Ontology (http://www.geneontology.org/).

## Supporting Information

Figure S1 **Mature miRNAs in hybrid yellow poplar were cloned by using stem-loop RT-PCR.** The mature miRNA sequence generated by deep sequencing technology is shown at the top. The corresponding sequence (underlined black line) of each trace file depicts the sequence result obtained by stem-loop RT-PCR.
(PDF)

Table S1 **Predicted conserved miRNAs detected by deep sequencing in hybrid yellow poplar (** ***L.tulipifera×L. chinense*** **).**
(DOC)

Table S2 **Predicted species-specific miRNAs identified from hybrid yellow poplar.**
(DOC)

Table S3 **Supplement of conserved miRNAs detected in hybrid yellow poplar by microarray analysis.**
(DOC)

Table S4 **Target proposed function of conserved miRNAs in hybrid yellow poplar.**
(DOC)

Table S5 **Target proposed function of species-specific miRNAs in hybrid yellow poplar.**
(DOC)

Table S6 **The mature miRNA sequences and primers used for RT-PCR and qRT-PCR.**
(DOC)
