# Supplementary material for: Deep Sequencing and Microarray Hybridization Identify Conserved and Species-Specific MicroRNAs during Somatic Embryogenesis in Hybrid Yellow Poplar
Source: PLoS One. 2012 Aug 29;7(8):e43451. doi: 10.1371/journal.pone.0043451 (PMC3430688; doi:10.1371/journal.pone.0043451)

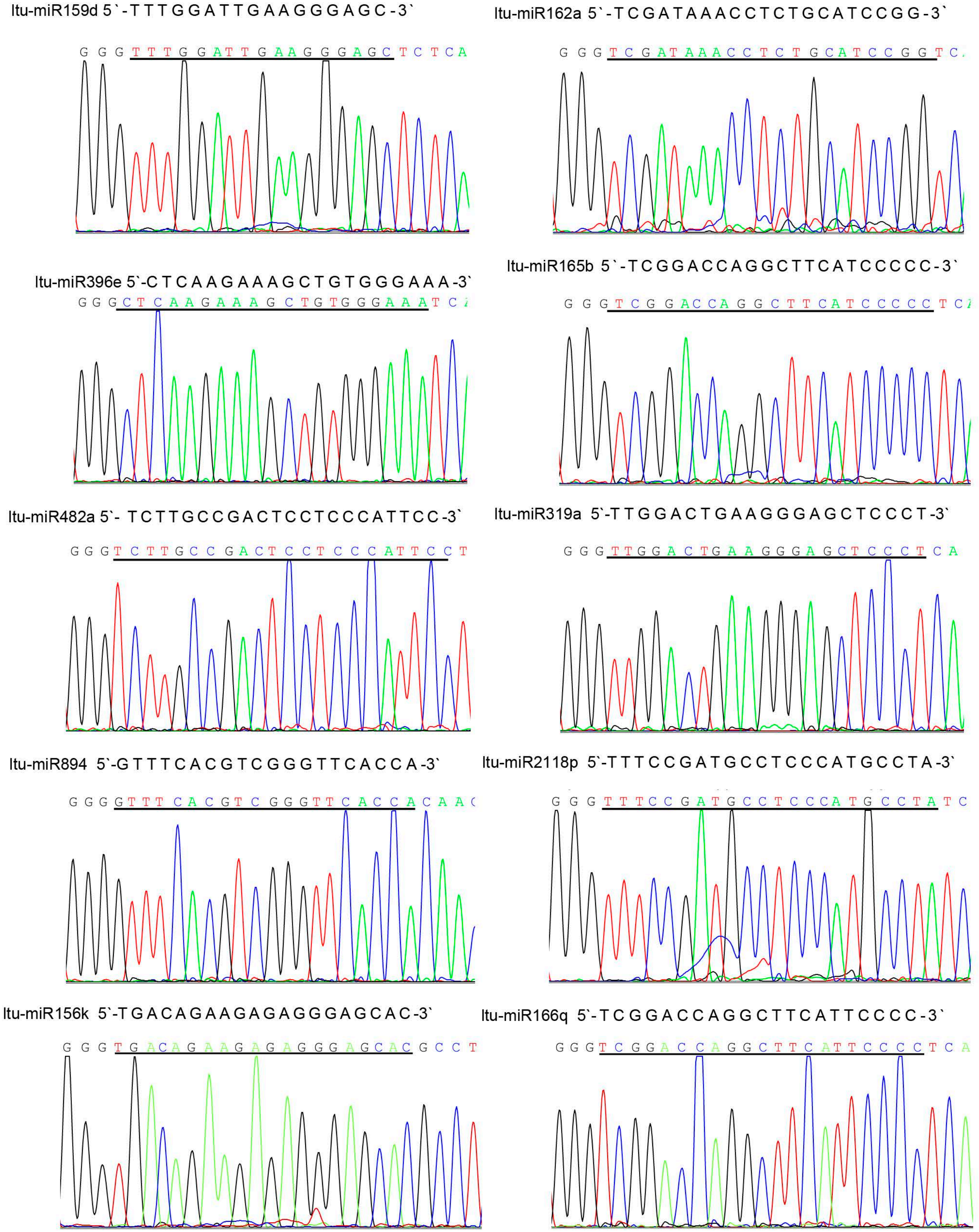
ltu-miR159d 5`-TTTGGATTGAAGGGAGC-3`
ltu-miR162a 5`-TCGATAAACCTCTGCATCCGG-3`
ltu-miR396e 5`-CTCAAGAAAGCTGTGGGAAA-3`
ltu-miR165b 5`-TCGGACCAGGCTTCATCCCCC-3`
ltu-miR482a 5`- TCTTGCCGACTCCTCCCATTCC-3`
ltu-miR319a 5`-TTGGACTGAAGGGAGCTCCCT-3`
ltu-miR894 5`-GTTTCACGTCGGGTTCACCA-3`
ltu-miR2118p 5`-TTTCCGATGCCTCCCATGCCTA-3`
ltu-miR156k 5`-TGACAGAAGAGAGGGAGCAC-3`
ltu-miR166q 5`-TCGGACCAGGCTTCATTCCCC-3`

ltu-miR390d 5`-A A G C T C A G G A G G G A T A G C G C C -3`

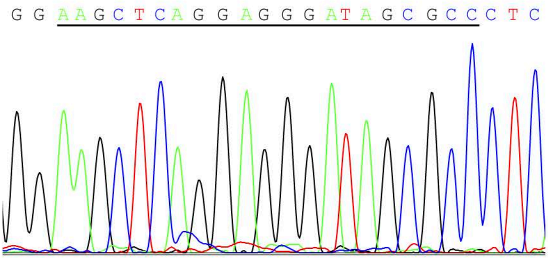

ltu-miR397b 5`-T C A T T G A G T G C A G C G T T G A T G -3 `

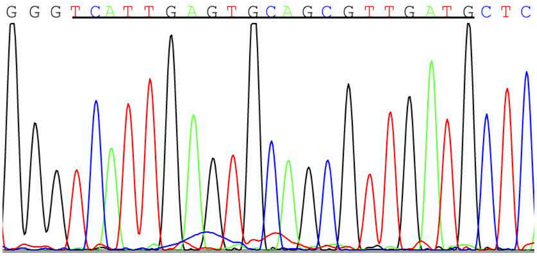

ltu-miRn1 5`-A T C T C T G A C A G C G G C A C G T G G C C C -3`

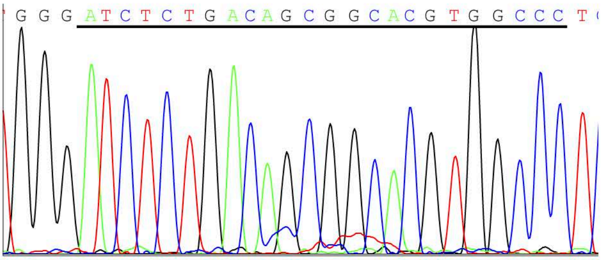

ltu-miRn3 5`-T T C C C A A T T C C T C C C A T G C C G T -3`

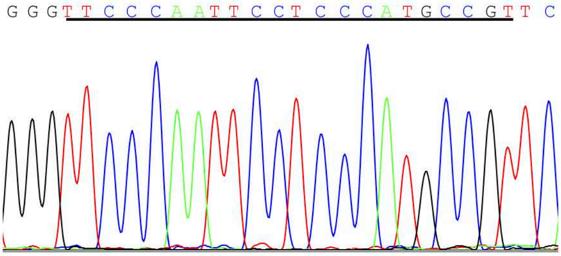

ltu-miRn4 5`-T T T C A A C A C T G A G G T C A T G G G -3`

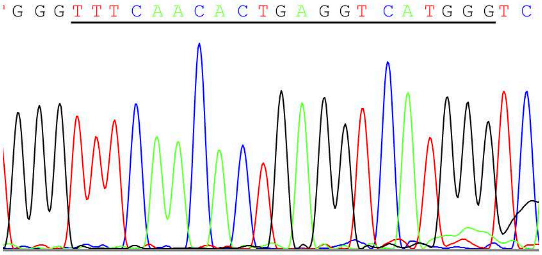

ltu-miR5 5`-T T C C C C A A G C C T C C C A T G C C G A -3`

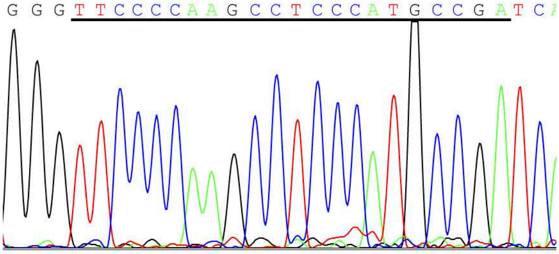

ltu-miRn8 5`-T T A G A C G A C T C T C G G C A A C -3`

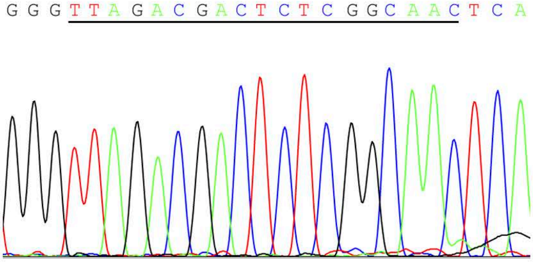

ltu-miRn86 5`-T T T G G A T C T G C C T C A T T T T T G -3`

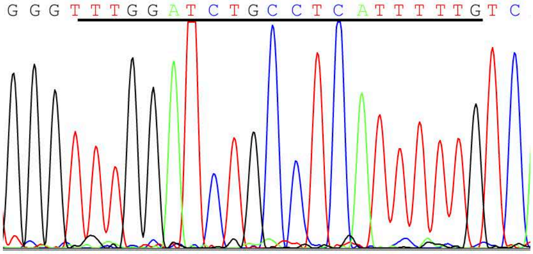

Supplement: Figure S1 — Mature miRNAs in hybrid yellow poplar were cloned by using stem-loop RT-PCR. The mature miRNA sequence generated by deep sequencing technology is shown at the top. The corresponding sequence (underlined black line) of each trace file depicts the sequence result obtained by stem-loop RT-PCR. (PDF) [file pone.0043451.s001.pdf]
