# Supplementary material for: Deep Sequencing and Microarray Hybridization Identify Conserved and Species-Specific MicroRNAs during Somatic Embryogenesis in Hybrid Yellow Poplar
Source: PLoS One. 2012 Aug 29;7(8):e43451. doi: 10.1371/journal.pone.0043451 (PMC3430688; doi:10.1371/journal.pone.0043451)
Supplement: Table S1 — Predicted conserved miRNAs detected by deep sequencing in hybrid yellow poplar ( L.tulipifera×L. chinense ). (DOC) [file pone.0043451.s002.doc]

## Table S1. Predicted conserved miRNAs detected by deep sequencing in hybrid yellow poplar (*L. tulipifera × L. chinense*).

|  |  |  |  |  |  | | | **Conserved in other plants^a^** | | | |  |
| --- | --- | --- | --- | --- | --- | --- | --- | --- | --- | --- | --- | --- |
| **No.** | **miRNA family** | **Name** | **Sequence (5′-3′)** | **Length(nt)** | | **Reference miRNA** | **ath** | | **ptc** | **vvi** | **osa** | **Reads** |
| 1 | 156 | ltu-MIR156f | UGACAGAAGAGAGUGAGCAC | 20 | | ptc-miR156f | ++ | | ++ | ++ | ++ | 4,750,907 |
| 2 |  | ltu-MIR156b | UUGACAGAAGAUAGAGAGCAC | 21 | | ahy-miR156b | ++ | | ++ | ++ | + | 39,026 |
| 3 |  | ltu-MIR156d | UGACAGAAGAGAGAGAGCAC | 20 | | mtr-miR156 | **−** | | + | + | ++ | 6,836 |
| 4 |  | ltu-MIR156j | CUGACAGAAGAGAGAGAGCAC | 21 | | ptc-miR156j | **−** | | + | + | ++ | 4,252 |
| 5 |  | ltu-MIR156e | UGACAGACGAGAGUGAGCAC | 20 | | vvi-miR156e | + | | + | + | + | 3,659 |
| 6 |  | ltu-MIR156k | UGACAGAAGAGAGGGAGCAC | 20 | | ptc-miR156k | + | | ++ | + | + | 3,369 |
| 7 |  | ltu-MIR156c | UGGCAGAAGAGAGUGAGCAC | 20 | | ghr-miR156c | + | | + | + | + | 3,304 |
| 8 |  | ltu-MIR156a | UGACAGAAGAUAGAGAGCAC | 20 | | pta-miR156a | ++ | | ++ | ++ | ++ | 1,673 |
| 9 |  | ltu-MIR156h | UGACAGAAGAAAGUGAGCAC | 20 | | ath-miR156h | + | | + | + | + | 1,103 |
| 10 |  | ltu-MIR156m | UGACAGAAGACAGUGAGCAC | 20 | | smo-MIR156d | + | | + | + | + | 370 |
| 11 |  | ltu-MIR156i | AGACAGAAGAGAGAGAGCAC | 20 | | ahy-miR156a | **−** | | + | + | + | 83 |
| 12 |  | ltu-MIR156l | UGAGAGAAGAGAGAGAGCAC | 20 | | ahy-miR156c | **−** | | + | + | + | 59 |
| 13 |  | ltu-MIR156g | UGACAGAAGAGAGAGGGCAC | 20 | | mtr-miR156g | **−** | | + | + | + | 47 |
| 14 | 159 | ltu-MIR159d | UUUGGAUUGAAGGGAGC | 17 | | ptc-miR159d | + | | + | + | + | 23 |
| 15 | 160 | ltu-MIR160d | UGCCUGGCUCCCUGUAUGCCA | 21 | | ptc-miR160d | ++ | | ++ | ++ | ++ | 139 |
| 16 |  | ltu-MIR160 | GCGTGCGAGGAGCCAAGCATA | 21 | | ptc-miR160 | − | | + | − | − | 8 |
| 17 | 162 | ltu-MIR162a | UCGAUAAACCUCUGCAUCCGG | 21 | | ptc-miR162a | + | | + | + | + | 1,859 |
| 18 | 164 | ltu-MIR164e | UGGAGAAGCAGGGCACGUGCA | 21 | | ptc-miR164e | ++ | | ++ | ++ | ++ | 23 |
| 19 | 166/165 | ltu-MIR165b | UCGGACCAGGCUUCAUCCCCC | 21 | | ath-miR165b | ++ | | + | + | + | 89 |
| 20 |  | ltu-MIR166q | UCGGACCAGGCUUCAUUCCCC | 21 | | ptc-miR166q | ++ | | ++ | ++ | ++ | 216,840 |
| 21 |  | ltu-MIR166d | UCGGGCCAGGCUUCAUUCCCC | 21 | | mtr-miR166d | + | | + | + | + | 139 |
| 22 |  | ltu-MIR166i | UCGGAUCAGGCUUCAUUCCCC | 21 | | osa-miR166i | + | | + | + | + | 137 |
| 23 |  | ltu-MIR166e | UCGAACCAGGCUUCAUUCCCC | 21 | | osa-miR166e | + | | + | + | ++ | 94 |
| 24 |  | ltu-MIR166k | UCGGACCAGGCUUCAAUCCCC | 21 | | zma-miR166k | + | | + | + | ++ | 27 |
| 25 |  | ltu-MIR166m | UCGGACCAGGCUUCAUUCCUUU | 22 | | ppt-miR166m | **−** | | ++ | + | ++ | 10 |
| 26 | 167 | ltu-MIR167e | UGAAGCUGCCAGCAUGAUCUA | 21 | | ptc-miR167e | ++ | | ++ | ++ | ++ | 14,546 |
| 27 |  | ltu-MIR167f | UGAAGCUGCCAGCAUGAUCUU | 21 | | ptc-MIR167f | ++ | | ++ | ++ | ++ | 81 |
| 28 |  | ltu-MIR167h | UGAAGCUGCCAACAUGAUCUA | 21 | | ptc-miR167h | + | | ++ | + | + | 11 |
| 29 |  | ltu-MIR167 | AGGUCAUGUGGCAGUUUCACC | 21 | | ahy-miR167-3p | **−** | | **−** | **−** | **−** | 3 |
| 30 | 168 | ltu-MIR168a | UCGCUUGGUGCAGGUCGGGAA | 21 | | ptc-miR168a | ++ | | ++ | ++ | + | 3,737 |
| 31 |  | ltu-MIR168c | UCGCUUGGUGCAGAUCGGGAC | 21 | | sbi-miR168 | + | | + | + | ++ | 117 |
| 32 |  | ltu-MIR168b | CCCGCCUUGCAUCAACUGAAU | 21 | | ptc-miR168b | **−** | | **−** | **−** | + | 18 |
| 33 |  | ltu-MIR168d | CCCGCCUUGCAUCAAGUGAAU | 21 | | zma-miR168b | + | | + | + | + | 12 |
| 34 |  | ltu-MIR168e | UUGCUUGGUGCAGGUCGGGAA | 21 | | mtr-miR168 | + | | + | + | + | 3 |
| 35 | 169 | ltu-MIR169s | CAGCCAAGGAUGACUUGCCGG | 21 | | ptc-miR169s | ++ | | ++ | ++ | ++ | 47 |
| 36 |  | ltu-MI169d | UGGCAAGUUGUCUUUGGCUAC | 21 | | vvi-miR169d | − | | + | + | + | 28 |
| 37 | 170 | ltu-MIR170 | UGAUUGAGCCGUGUCAAUAUC | 20 | | ath-miR170 | ++ | | + | + | + | 3 |
| 38 | 171 | ltu-MIR171i | UGAUUGAGCCGUGCCAAUAUC | 21 | | ptc-miR171i | + | | ++ | ++ | ++ | 857 |
| 39 |  | ltu-MIR171d | UUGAGCCGCGCCAAUAUCACU | 21 | | sly-miR171d | **−** | | + | ++ | + | 459 |
| 40 |  | ltu-MIR171b | UUGAGCCGCGUCAAUAUCUCC | 21 | | osa-miR171i | + | | + | + | + | 348 |
| 41 |  | ltu-MIR171f | CGAUGUUGGUGAGGUUCAAUC | 21 | | vvi-miR171f | + | | + | + | + | 165 |
| 42 |  | ltu-MIR171a | UUGAGCCGCGCCAAUAUCAC | 20 | | ptc-miR171d | + | | + | ++ | + | 13 |
| 43 | 172 | ltu-MIR172e | GGAAUCUUGAUGAUGCUGCAU | 21 | | ptc-miR172e | ++ | | ++ | ++ | ++ | 13 |
| 44 | 319 | ltu-MIR319a | UUGGACUGAAGGGAGCUCCCU | 21 | | smo-miR319 | ++ | | ++ | ++ | + | 12 |
| 45 |  | ltu-MIR319b | UUGGACUGAAGGGAGCUCCC | 20 | | ptc-miR319b | ++ | | ++ | ++ | + | 4 |
| 46 | 390 | ltu-MIR390d | AAGCUCAGGAGGGAUAGCGCC | 21 | | ptc-miR390d | ++ | | ++ | ++ | ++ | 2,768 |
| 47 |  | ltu-MIR390b | AAGCUCAGGAUGGAUAGCGCC | 21 | | pta-miR390 | + | | + | + | + | 8 |
| 48 |  | ltu-MIR390a | CGCUAUCCAUCCUGAGUUU | 19 | | gma-miR390a | + | | + | + | + | 5 |
| 49 | 394 | ltu-MIR394a | UUGGCAUUCUGUCCACCUCC | 20 | | ghr-miR394a | ++ | | ++ | ++ | ++ | 100 |
| 50 | 396 | ltu-MIR396e | CUCAAGAAAGCUGUGGGAAA | 21 | | ptc-miR396e | + | | + | + | + | 15,514 |
| 51 |  | ltu-MIR396g | UCAGUACAAAGCUCAAGAAAG | 21 | | ptc-MIR396g | + | | + | + | + | 673 |
| 52 |  | ltu-MIR396g | UUCCACGGCUUUCUUGAACUU | 21 | | ptc-miR396g | + | | ++ | + | + | 156 |
| 53 |  | ltu-MIR396 | UCCACAGGCUUUCUUGAACUG | 21 | | sbi-miR396e | + | | + | + | ++ | 3 |
| 54 | 397 | ltu-MIR397b | UCAUUGAGUGCAGCGUUGAUG | 21 | | ptc-miR397b | ++ | | ++ | ++ | ++ | 8,860 |
| 55 |  | ltu-MIR397d | UCAUUGAGUGCAUCGUUGAUG | 21 | | ath-miR397b | ++ | | + | + | + | 77 |
| 56 |  | ltu-MIR397c | UCAUUGAGUGCAGCUUUGAUG | 21 | | ptc-miR397c | + | | + | + | + | 52 |
| 57 | 398 | ltu-MIR398b | UGUGUUCUCAGGUCGCCCCUG | 21 | | ptc-miR398b | **−** | | ++ | ++ | ++ | 525 |
| 58 |  | ltu-MIR398a | UGUGUUCUCAGGUCGCCCCUU | 21 | | ptc-miR398a | + | | ++ | ++ | ++ | 15 |
| 59 | 399 | ltu-MIR399j | UGCCAAAGGAGAUUUGUCCGG | 21 | | ptc-miR399j | + | | ++ | + | + | 122 |
| 60 |  | ltu-MIR399b | UGCCAAAGGAGAUUUGCCCGG | 21 | | ptc-miR399b | ++ | | ++ | ++ | + | 76 |
| 61 |  | ltu-MIR399l | CGCCAAAGGAGAGUUGCCCUA | 21 | | ptc-miR399l | + | | ++ | ++ | ++ | 10 |
| 62 | 408 | ltu-MIR408b | CUGCACUGCCUCUUCCCUGGC | 21 | | sof-miR408b | ++ | | ++ | ++ | ++ | 809 |
| 63 | 472 | ltu-MIR472b | UUUCCAACUCCACCCAU | 17 | | ptc-miR472b | + | | + | + | **−** | 5 |
| 64 | 477 | ltu-MIR477 | AUCUCCCUCAAAGGCGUCCAA | 21 | | vvi-miR477 | **−** | | + | + | **−** | 22 |
| 65 | 482 | ltu-MIR482a | UCUUGCCGACUCCUCCCAUUCC | 21 | | aqc-miR482a | **−** | | + | + | + | 965 |
| 66 | 528 | ltu-MIR528a | UGGAAGGGGCAUGCAGAGGAG | 21 | | zma-miR528a | **−** | | **−** | **−** | ++ | 5 |
| 67 | 529 | ltu-MIR529d | AGAAGAGAGAGAGCACAGCCC | 21 | | ppt-miR529d | **−** | | + | + | + | 13 |
| 68 | 535 | ltu-MIR535a | UGACAACGAGAGAGAGCACGC | 21 | | ppt-miR535a | **−** | | **−** | ++ | ++ | 30,916 |
| 69 | 827 | ltu-MIR827b | UUAGAUGAUCAUCAACAAACA | 21 | | ghr-miR827b | + | | + | **−** | + | 382 |
| 70 | 894 | ltu-MIR894 | GUUUCACGUCGGGUUCACCA | 20 | | ppt-miR894 | **−** | | **−** | **−** | **−** | 318 |
| 71 | 1432 | ltu-MIR1432 | UCAGGAGAGAUGACACCGACG | 21 | | sbi-miR1432 | + | | **−** | + | ++ | 3 |
| 72 | 1510a | ltu-MIR1510 | UUUUACCUAUUCCACCCAUUCC | 22 | | gma-miR1510a | − | | − | − | − | 18 |
| 73 | 1511 | ltu-MIR1511 | AACCUGGCUCUGAUACCA | 18 | | gma-miR1511 | **−** | | **−** | **−** | **−** | 27 |
| 74 | 2118 | ltu-MIR2118p | UUUCCGAUGCCUCCCAUGCCUA | 22 | | osa-miR2118p | **−** | | **−** | **−** | ++ | 18 |
| 75 | 2911 | ltu-MIR2911 | CGGGGGACGGACUGGGAACGGCCCCC | 26 | | peu-miR2911 | − | | − | − | − | 92 |

^a^ath, *Arabidopsis thaliana*; ptc, *Populus trichocarpa*; vvi, *Vitis vinifera*; osa, *Oryza sativa*; ++, miRNA sequences of hybrid yellow poplar were identical to those in other species; +, miRNA sequences of hybrid *Liriodendron* were conserved in other species but varied at some nucleotide positions; **−**, miRNA sequences of hybrid yellow poplar could not be found in those species.
