# Supplementary material for: Deep Sequencing and Microarray Hybridization Identify Conserved and Species-Specific MicroRNAs during Somatic Embryogenesis in Hybrid Yellow Poplar
Source: PLoS One. 2012 Aug 29;7(8):e43451. doi: 10.1371/journal.pone.0043451 (PMC3430688; doi:10.1371/journal.pone.0043451)
Supplement: Table S2 — Predicted species-specific miRNAs identified from hybrid yellow poplar. (DOC) [file pone.0043451.s003.doc]

## Table S2. Predicted species-specific miRNAs identified from hybrid yellow poplar (*L. tulipifera × L. chinense*).

| **miRNA** | **Sequence (5′–3′)** | **Length** | **Isoform copy no.** | **Chr_Seq ID^a^** | **Strand** | **Location** | **Hairpin length** | **dG^b^** | **CG%^c^** | **MFEI^d^** |
| --- | --- | --- | --- | --- | --- | --- | --- | --- | --- | --- |
| ltu-miRn1 | AUCUCUGACAGCGGCACGUGGCCC | 24 | 10109 | DT582597 | + | 3’ | 198 | −106.6 | 54.4 | 1 |
|  |  | 24 | 10109 | FD487496 | − | 3′ | 198 | −106.6 | 53.9 | 1 |
| ltu-miRn2 | GAGGUGGAAUCUCUUGGCGUCUUAU | 25 | 3202 | DT584429 | + | 3′ | 117 | −48.8 | 53.7 | 0.7 |
| ltu-miRn3 | UUCCCAAUUCCUCCCAUGCCGU | 22 | 3135 | FD499227 | + | 3′ | 146 | −62 | 44.1 | 0.9 |
|  |  | 22 | 3135 | FD495530 | + | 3′ | 116 | −53 | 45.1 | 1 |
|  |  | 22 | 3135 | FD495932 | + | 3′ | 146 | −60.1 | 44.7 | 0.9 |
|  |  | 22 | 3135 | FD492514 | + | 3′ | 146 | −62 | 44.1 | 0.9 |
|  |  | 22 | 3135 | DT595608 | + | 3′ | 116 | −53 | 45.1 | 1 |
|  |  | 22 | 3135 | CK758787 | + | 3′ | 188 | −70.4 | 45.4 | 0.8 |
| ltu-miRn4 | UUUCAACACUGAGGUCAUGGG | 21 | 1337 | FD488622 | + | 3′ | 51 | −26.4 | 56.1 | 0.8 |
|  |  | 21 | 1337 | FD501889 | − | 3′ | 51 | −26.4 | 56.1 | 0.8 |
|  |  | 21 | 1337 | DT597278 | − | 3′ | 51 | −26.6 | 54.4 | 0.9 |
| ltu-miRn5 | UUCCCCAAGCCUCCCAUGCCGA | 22 | 543 | CK758787 | + | 5′ | 193 | −61.7 | 45.2 | 0.7 |
| ltu-miRn6 | UGGAUUCCAAGAGCAUCUCU | 20 | 239 | DT584429 | + | 3′ | 147 | −68.9 | 48.7 | 0.9 |
| ltu-miRn7 | UGGAUUCCGAGAGCUCCUCCC | 21 | 192 | FD499227 | + | 3′ | 135 | −56.3 | 49.6 | 0.8 |
|  |  | 21 | 192 | FD495932 | + | 3′ | 134 | −57.3 | 50.7 | 0.8 |
|  |  | 21 | 192 | DT595608 | + | 3′ | 135 | −56.3 | 49.6 | 0.8 |
|  |  | 21 | 192 | DT584429 | + | 3′ | 139 | −59.6 | 51.7 | 0.8 |
|  |  | 21 | 192 | CK750940 | + | 3′ | 135 | −56.3 | 49.6 | 0.8 |
| ltu-miRn8 | UUAGACGACUCUCGGCAAC | 19 | 180 | FD491465 | − | 3′ | 82 | −34.4 | 64 | 0.6 |
| ltu-miRn9 | AUCGAAGUAGAUGAAUGUGAC | 21 | 169 | FD501285 | − | 5′ | 82 | −26.3 | 29.5 | 1 |
| ltu-miRn10 | AGGCUGAGUUGGACCAAUCAG | 21 | 151 | DU169578 | + | 3′ | 72 | −50.6 | 48.7 | 1.3 |
| ltu-miRn11 | CAUGGCAGUAGAAGAGAUCACGUA | 24 | 126 | CK755170 | + | 5′ | 81 | −43.1 | 43.5 | 1.2 |
| ltu-miRn12 | ACCAUCAUUGGAGCGCAUUGGACA | 24 | 69 | FD492501 | + | 3′ | 154 | −41.6 | 42.5 | 0.6 |
| ltu-miRn13 | AUGACAUGGUGAGAUUUGAUUGGU | 24 | 58 | DU169538 | + | 5′ | 75 | −37.6 | 45.6 | 1 |
| ltu-miRn14 | CGCUGCUGACUCGUUGGCUCA | 21 | 43 | CK760683 | + | 5′ | 207 | −97.1 | 51.6 | 0.9 |
| ltu-miRn15 | ACAAUGGAAUUGUAAUCAAUGGAC | 24 | 43 | DT581764 | + | 3′ | 135 | −32.8 | 33.3 | 0.7 |
| ltu-miRn16 | CCAGUCGUGACCGGUCGGCCA | 21 | 42 | DU169650 | − | 3′ | 105 | −51.2 | 57.7 | 0.8 |
| ltu-miRn17 | CGAGUGGGAGAAGGUACUCAA | 21 | 36 | FD500253 | + | 5′ | 118 | −27.4 | 38.7 | 0.6 |
| ltu-miRn18 | AUUUCGAUUGUUUGGAUGCUC | 21 | 34 | DT579782 | − | 5′ | 237 | −60.1 | 36.9 | 0.7 |
| ltu-miRn19 | AGGAUUCGUGGGCUGAGAUCACGG | 24 | 31 | DU169564 | − | 5′ | 77 | −34.4 | 56.6 | 0.7 |
| ltu-miRn20 | UGUCACGUAAGUAGGAUCGUC | 21 | 30 | FD496450 | − | 5′ | 69 | −19.2 | 39 | 0.6 |
|  |  | 21 | 30 | FD496447 | − | 5′ | 50 | −15.5 | 41.1 | 0.7 |
| ltu-miRn21 | AUUUGUAGAAGUCGAGGCGUGAUU | 24 | 28 | FD498773 | + | 5′ | 51 | −16.3 | 38.6 | 0.7 |
| ltu-miRn22 | AAAGGGUUUACAUGCAAUUAGAGG | 24 | 26 | FD494232 | + | 5′ | 218 | −84.1 | 31.2 | 1.2 |
| ltu-miRn23 | AGAGCCUUUGUGAAGAUAUCUGCUA | 25 | 24 | DT600953 | + | 3′ | 54 | −12.7 | 43.1 | 0.5 |
| ltu-miRn24 | GACAGAUAGAGAGAAAGCAA | 20 | 24 | FD499019 | − | 3′ | 129 | −22.853 | 43.1 | 0.4 |
| ltu-miRn25 | UAGUAGUAACCAUUAAUCUAC | 21 | 24 | FD501285 | − | 3′ | 82 | −26.3 | 29.5 | 1 |
| ltu-miRn26 | AACAGUUUGGAUGGCAAAUAAACA | 24 | 24 | FD499501 | − | 3′ | 245 | −73.8 | 41 | 0.7 |
| ltu-miRn27 | CAAGUCAUGGUAGCUCAGGGC | 21 | 23 | CK762472 | − | 5′ | 74 | −21.6 | 45.9 | 0.6 |
| ltu-miRn28 | AGGCGACGGGAUCUUGGGUUCAUC | 24 | 21 | DU169441 | + | 3′ | 128 | −47.7 | 55.2 | 0.6 |
| ltu-miRn29 | AGGGACUCUGUGUGCGCAGGGAGG | 24 | 19 | FD497590 | − | 3′ | 55 | −23.7 | 45.8 | 0.9 |
| ltu-miRn30 | CGUUCAUUGUACGGAUCUAGGGAC | 24 | 19 | CK745828 | + | 3′ | 63 | −32.2 | 59.4 | 0.8 |
| ltu-miRn31 | UCGCAUUGGAAGAGGAUGUUA | 21 | 19 | FD495154 | + | 3′ | 117 | −25.9 | 33.3 | 0.6 |
|  |  | 21 | 19 | CK751931 | + | 3′ | 117 | −25.9 | 33.3 | 0.6 |
| ltu-miRn32 | UGAUCGCUUCUAUUGUUAUUUC | 22 | 18 | CK755170 | + | 3′ | 81 | −43.1 | 43.5 | 1.2 |
| ltu-miRn33 | AUCUAAUGGUUGGAGUGGAUUUCA | 24 | 17 | FD493586 | − | 3′ | 221 | −78.6 | 44.6 | 0.8 |
| ltu-miRn34 | GAUGGGUUUAGGUGGUAUGGAAUU | 24 | 16 | CK747472 | + | 5′ | 52 | −16.2 | 42.4 | 0.6 |
| ltu-miRn35 | CAAAGAUCACUGCAAUCGCCAA | 22 | 16 | CK755529 | + | 5′ | 231 | −46.6 | 37.1 | 0.5 |
| ltu-miRn36 | GACCUGGCAGCUGACAUUU | 19 | 16 | FD499873 | + | 3′ | 86 | −29.1 | 39.1 | 0.8 |
|  |  | 19 | 16 | FD491293 | + | 3′ | 86 | −29.1 | 39.1 | 0.8 |
| ltu-miRn37 | ACAACGUAGAAGAUUUCCAAGACU | 24 | 16 | FD501875 | − | 3′ | 77 | −14.2 | 36.1 | 0.5 |
| ltu-miRn38 | AGGAUUUGAUUGGGCCACGUGCU | 23 | 15 | DT582597 | + | 5′ | 157 | −88.6 | 53.1 | 1 |
|  |  | 23 | 15 | FD487496 | − | 5′ | 243 | −116.8 | 54.6 | 0.9 |
| ltu-miRn39 | AGCCCAAAAAUGAGGCAGAUCCGA | 24 | 15 | DT598237 | + | 5′ | 172 | −81.6 | 44.4 | 1 |
| ltu-miRn40 | UAGGAAGUUUUUAAUGGUGGACGU | 24 | 14 | DU169578 | + | 3′ | 145 | −55.3 | 37.6 | 1 |
| ltu-miRn41 | CCCACUUGAGCGUUGGAUCUG | 21 | 13 | DT581323 | + | 3′ | 132 | −56.4 | 44.2 | 0.9 |
|  |  | 21 | 13 | DT580670 | + | 3′ | 137 | −58.9 | 44.9 | 1 |
| ltu-miRn42 | AUCCAAUCGGCUUCAAGGGGUAAU | 24 | 13 | CK763735 | − | 3′ | 64 | −21.1 | 47.1 | 0.6 |
| ltu-miRn43 | AAAUUCAUCUGACAGCCCGAUACA | 24 | 12 | CK752154 | + | 5′ | 110 | −22.2 | 34.5 | 0.6 |
| ltu-miRn44 | UCCAAAACUCAAGUGGACCAUGCC | 24 | 12 | DT584335 | + | 5′ | 241 | −79.1 | 41.5 | 0.8 |
| ltu-miRn45 | UCCAAAACUCAAGUGAGCCACAU | 23 | 12 | FD493209 | − | 5′ | 84 | −29.6 | 37.8 | 0.9 |
|  |  | 23 | 12 | FD489763 | − | 5′ | 134 | −42.1 | 33.6 | 0.9 |
| ltu-miRn46 | AUAGACUACGCUACACGCUACAUA | 24 | 12 | CK764894 | + | 3′ | 84 | −21.4 | 41.1 | 0.6 |
| ltu-miRn47 | UAGUAGAUUUGAAAGUAGAUUA | 22 | 12 | FD501285 | + | 3′ | 16 | −28 | 34.6 | 0.8 |
| ltu-miRn48 | AGUGCGUCGAUCUGAUACCCGAGC | 24 | 12 | FD493790 | + | 3′ | 70 | −24.3 | 56.6 | 0.6 |
| ltu-miRn49 | UUAGAGCAAGUGGUUAGUCGUCCU | 24 | 12 | FD496230 | − | 3′ | 70 | −21.1 | 42.1 | 0.7 |
|  |  | 24 | 12 | CK748113 | − | 3′ | 59 | −25.7 | 49.2 | 0.8 |
| ltu-miRn50 | UAGAAUACAUACAUCAAGGUG | 21 | 11 | DU169645 | + | 5′ | 203 | −85.9 | 42.1 | 1 |
| ltu-miRn51 | AUGAGUGGGAGAAAGUGCUUA | 21 | 11 | CK762472 | + | 5′ | 130 | −36.3 | 44.1 | 0.6 |
| ltu-miRn52 | AGGCAACGUGUGAUCAUUGAUGAA | 24 | 11 | FD492016 | − | 5′ | 132 | −19.7 | 34.8 | 0.4 |
| ltu-miRn53 | CCGGUCUUGACCGGUCGGCCA | 21 | 11 | FD498392 | + | 5′ | 60 | −40.6 | 62.1 | 1 |
|  |  | 21 | 11 | FD488305 | + | 5′ | 66 | −48.7 | 65.2 | 1.1 |
|  |  | 21 | 11 | DU169660 | − | 5′ | 110 | −83.2 | 64.7 | 1.1 |
|  |  | 21 | 11 | FD496975 | − | 5′ | 70 | −38 | 55.3 | 0.9 |
| ltu-miRn54 | AGAGGUCUAGGUCUCGAAACCCAU | 24 | 11 | FD490601 | + | 3′ | 71 | −38.7 | 49.4 | 1 |
| ltu-miRn55 | CAUUAGAAGAUAGAAGUCACAU | 22 | 11 | FD501285 | + | 3′ | 82 | −31 | 29.5 | 1.2 |
| ltu-miRn56 | GGCUUGGAGGUAUAUUGGGAAGG | 23 | 10 | FD495530 | + | 5′ | 116 | −53 | 45.1 | 1 |
|  |  | 23 | 10 | FD495932 | + | 5′ | 167 | −67.8 | 46.2 | 0.8 |
|  |  | 23 | 10 | FD492514 | + | 5′ | 188 | −72.3 | 44.8 | 0.8 |
|  |  | 23 | 10 | DT599746 | + | 5′ | 116 | −51.1 | 45.9 | 0.9 |
|  |  | 23 | 10 | DT595608 | + | 5′ | 167 | −69.7 | 46.2 | 0.9 |
|  |  | 23 | 10 | CK758787 | + | 5′ | 167 | −67.8 | 46.8 | 0.8 |
| ltu-miRn57 | ACGUCGCAGAUCGAGUGUUGGAGC | 24 | 10 | DU169365 | − | 5′ | 197 | −53.3 | 49.3 | 0.5 |
| ltu-miRn58 | CCUAGGAAGUUUUUAAUGGUGGAC | 24 | 10 | DU169684 | + | 3′ | 82 | −23.1 | 33 | 0.8 |
| ltu-miRn59 | AGAAUAUGCACAAUUAAGAGCUCA | 24 | 10 | DU169666 | + | 3′ | 150 | −34.4 | 36.8 | 0.6 |
| ltu-miRn60 | AGAAUUAGCUUCGGGUAGCUUGGG | 24 | 10 | DT583410 | − | 3′ | 111 | −38.6 | 46.9 | 0.7 |
| ltu-miRn61 | AAUUGAAUGACUUGUCCGACAAUC | 24 | 10 | DU169439 | − | 3′ | 91 | −20.3 | 37 | 0.6 |
| ltu-miRn62 | AAGCUGCAAAGAUCAUGAAAGAAA | 24 | 10 | DT583583 | + | 3′ | 75 | −16.2 | 42 | 0.5 |
| ltu-miRn63 | CGAGUUGAUGUGGAUGGACGCCGG | 24 | 10 | FD495445 | − | 3′ | 53 | −21.2 | 63.3 | 0.6 |
| ltu-miRn64 | CGGGCCGAGAGUGGAACGGGG | 21 | 9 | FD488523 | + | 5′ | 116 | −59.4 | 65.5 | 0.8 |
| ltu-miRn65 | AGGUCUCUCAUAACUCAAUCGGAA | 24 | 9 | DU169517 | − | 5′ | 57 | −15.9 | 42.6 | 0.6 |
| ltu-miRn66 | UGCUCUGAUACCAUGUUAAA | 20 | 9 | FD491377 | − | 5′ | 82 | −51.8 | 39.8 | 1.6 |
| ltu-miRn67 | AAUGAAAAAGUGUCAUGUGCGCCU | 24 | 9 | DT580988 | + | 5′ | 139 | −57.4 | 46.2 | 0.9 |
| ltu-miRn68 | AUGGAUUUGAUUCGCAUGCUCCUG | 24 | 9 | FD491688 | − | 5′ | 112 | −34.3 | 51.7 | 0.6 |
| ltu-miRn69 | CUGUUUUCUGUGGUGUGGUCC | 21 | 9 | CK766565 | + | 5′ | 125 | −47.1 | 46.6 | 0.8 |
| ltu-miRn70 | AGCCAUGAGAACCGGAUCCAAACU | 24 | 9 | FD497713 | − | 5′ | 103 | −31.6 | 44 | 0.7 |
| ltu-miRn71 | AGUGGUAUGGCAUGUCAGAAAACC | 24 | 9 | FD491756 | − | 3′ | 80 | −32.3 | 45.3 | 0.8 |
| ltu-miRn72 | AAGUGGAAAUUGGAGGGCCAAGCC | 24 | 9 | FD498484 | − | 3′ | 142 | −43.8 | 40.5 | 0.7 |
| ltu-miRn73 | GUCACGUAAGUAGGAUCGUCG | 21 | 8 | FD495154 | + | 5′ | 50 | −15.5 | 41.1 | 0.7 |
|  |  | 21 | 8 | FD487843 | + | 5′ | 26 | −16.1 | 43.2 | 0.4 |
|  |  | 21 | 8 | CK751931 | + | 5′ | 50 | −15.5 | 41.1 | 0.7 |
| ltu-miRn74 | AUUCUCAGAAGAAACUCAACCCAA | 24 | 8 | CV005845 | + | 5′ | 57 | −17.3 | 36.9 | 0.7 |
| ltu-miRn75 | GAAGAUCUAAUGCUCAAGUCGGCU | 24 | 8 | DT581323 | + | 5′ | 138 | −60.2 | 45.8 | 0.9 |
|  |  | 24 | 8 | DT580670 | + | 5′ | 137 | −58.9 | 44.9 | 1 |
| ltu-miRn76 | AUUUCAGAUUUUUUGAACGGUUUA | 24 | 8 | DU169386 | + | 3′ | 86 | −17.4 | 37 | 0.5 |
| ltu-miRn77 | CACAUGGCCCAAUCAAAUCCUGCC | 24 | 8 | FD487496 | + | 3′ | 157 | −89.1 | 53.1 | 1 |
|  |  | 24 | 8 | DT586339 | + | 3′ | 157 | −89.1 | 53.1 | 1 |
| ltu-miRn78 | CCGGUCUUGACCGGUCGGGCA | 21 | 8 | FD493308 | − | 3′ | 80 | −43.5 | 59.3 | 0.9 |
| ltu-miRn79 | ACAAACAAGUGGACAAGCUGAGAU | 24 | 8 | DU169511 | − | 3′ | 116 | −29.9 | 43.4 | 0.6 |
| ltu-miRn80 | UCGAGAAAUGACUAUAGCUAGGAG | 24 | 8 | FD495347 | − | 3′ | 64 | −18.4 | 45.8 | 0.6 |
| ltu-miRn81 | CGAGCCGCGCGGGGCCGCCUUG | 22 | 8 | DT586282 | + | 3′ | 54 | −32.6 | 70 | 0.8 |
| ltu-miRn82 | CGGCGGCUGCUGCGAAACCU | 20 | 7 | FD500799 | + | 5′ | 55 | −37 | 76.8 | 0.9 |
| ltu-miRn83 | UAGCAGAAGGAUUCGUGCAGA | 21 | 7 | CK762472 | + | 3′ | 51 | −9.5 | 44.4 | 0.4 |
| ltu-miRn84 | CUUAUGAACAGGUUGGAUGGCAAA | 24 | 7 | DU169372 | + | 3′ | 54 | −16.7 | 31.7 | 0.9 |
| ltu-miRn85 | GGAUGGAAGGGCAGAGGCCUUUGGU | 25 | 7 | NC_008326 | + | 3′ | 61 | −32.3 | 52.2 | 0.9 |
|  |  | 25 | 7 | NC_008326 | − | 3′ | 61 | −32.3 | 52.2 | 0.9 |
| ltu-miRn86 | UUUGGAUCUGCCUCAUUUUUG | 21 | 7 | DU169505 | − | 3′ | 122 | −35.1 | 44.5 | 0.6 |
|  |  | 21 | 7 | FD500829 | − | 3′ | 243 | −85.9 | 37.8 | 0.9 |
| ltu-miRn87 | UUGGAAGAGGAUGUUAAGUUA | 21 | 7 | FD491555 | + | 3′ | 157 | −34.9 | 35.6 | 0.6 |
| ltu-miRn88 | UGUGCAAGUGGAGCCCACGCC | 21 | 7 | DT594845 | + | 3′ | 61 | −15.1 | 41.8 | 0.5 |
| ltu-miRn89 | AGGACUAUAUGAUUGUAUUGAAAG | 24 | 7 | FD501619 | − | 3′ | 66 | −15.8 | 27.8 | 0.8 |
| ltu-miRn90 | AGAGGCUGAUAUAUCCAGGAUUUG | 24 | 7 | DU169584 | − | 3′ | 253 | −71.9 | 46.5 | 0.6 |
| ltu-miRn91 | UAACGCGUCAGAAAAUGGGCC | 21 | 7 | FD496025 | + | 3′ | 59 | −19.9 | 55.4 | 0.6 |
| ltu-miRn92 | UCUCAGUGGAGUACUUUCGAA | 21 | 6 | FD496450 | − | 5′ | 196 | −46.9 | 37.7 | 0.6 |
| ltu-miRn93 | CAGAAAGCAACUAAGGGUGGCUGG | 24 | 6 | DU169439 | − | 5′ | 58 | −23.7 | 54.7 | 0.7 |
| ltu-miRn94 | AGAAGCUGCAAAGAUCAUGAAAGA | 24 | 6 | DT583583 | + | 5′ | 75 | −16.2 | 42 | 0.5 |
| ltu-miRn95 | UCCAGAACAUGCAACGUUGUC | 21 | 6 | FD501285 | + | 5′ | 77 | −27.5 | 37.3 | 0.9 |
| ltu-miRn96 | AUGGGCCAAGGGAUUGAGAGGGUU | 24 | 6 | DT583410 | − | 5′ | 170 | −62.5 | 46.6 | 0.7 |
| ltu-miRn97 | UCCUGUGGUGUGGUCCAUCUGA | 22 | 6 | DT598429 | + | 5′ | 109 | −35.9 | 42.9 | 0.7 |
| ltu-miRn98 | AAAACAAAACGAAUUCCUGCGGUA | 24 | 6 | CV005845 | − | 5′ | 101 | −23.8 | 39.3 | 0.6 |
| ltu-miRn99 | ACUUUUAAUGUGGAAGAUCUAGUC | 24 | 6 | DT583579 | + | 3′ | 63 | −23.8 | 43.5 | 0.8 |
| ltu-miRn100 | GGAACAAUUUUCGAUUGAUCGGAA | 24 | 6 | DU169617 | − | 3′ | 84 | −38.4 | 41.7 | 1.1 |
| ltu-miRn101 | AUACCAGGGUUUGUCAGGGACAGC | 24 | 6 | CK754544 | − | 3′ | 83 | −35.4 | 51.7 | 0.8 |
| ltu-miRn102 | CGGUCUUGACCGGUCGGCUGG | 21 | 6 | DU169603 | − | 3′ | 96 | −59.3 | 64.7 | 0.9 |
| ltu-miRn103 | CGGACAGUCUUCCGAUCCAUCGGA | 24 | 6 | DU169617 | + | 3′ | 118 | −66.3 | 48.4 | 0.9 |
|  |  | 24 | 6 | DU169625 | − | 3′ | 140 | −41.4 | 45.9 | 0.6 |
| ltu-miRn104 | CAUGUGGGCUAUGUGCAUCAACUU | 24 | 6 | DU169551 | − | 3′ | 110 | −48.7 | 43.1 | 1 |
| ltu-miRn105 | AAAUGGAUGGACAGCAUGGAU | 21 | 6 | CK766565 | + | 3′ | 97 | −36.5 | 44.7 | 0.8 |
| ltu-miRn106 | UGGACGGUGUGGAUAUAGCAC | 21 | 6 | FD501598 | − | 3′ | 252 | −70.2 | 36 | 0.8 |
| ltu-miRn107 | GUUCCGUAGAGCACCCCAGGUGGC | 24 | 6 | DU169337 | − | 3′ | 55 | −33.3 | 59 | 0.9 |
| ltu-miRn108 | AUUUGAGCUUUGGAUGUGCCUCAU | 24 | 6 | DT598237 | − | 3′ | 172 | −81 | 44.6 | 1 |
| ltu-miRn109 | AAUAGCUUAAGUUGCAUGUAGCCU | 24 | 6 | FD491843 | − | 3′ | 52 | −14.9 | 41.4 | 0.6 |
| ltu-miRn110 | AACUCCUGUGGCUAAAGGCUUUAG | 24 | 6 | FD498158 | − | 3′ | 93 | −53 | 44.4 | 1.2 |
| ltu-miRn111 | UCGUGGCGAAGACAUCAUUGAU | 22 | 6 | FD501625 | − | 3′ | 86 | −26.9 | 42.4 | 0.7 |
|  |  | 22 | 6 | FD495347 | − | 3′ | 64 | −18.4 | 45.8 | 0.6 |
|  |  | 22 | 6 | FD493822 | − | 3′ | 86 | −25.6 | 45.7 | 0.6 |
| ltu-miRn112 | AUACGAUGAGUGGGUUCCGAGUGA | 24 | 6 | DU169415 | − | 3′ | 79 | −29.9 | 50.6 | 0.7 |
| ltu-miRn113 | AUGGAAUCGGUGUGGUCCGAGGUG | 24 | 6 | DU169466 | − | 3′ | 130 | −36.7 | 48.5 | 0.6 |
| ltu-miRn114 | AGGCCAGAUCCAGCCAAUGAACGG | 24 | 5 | CK745828 | + | 5′ | 63 | −32.2 | 59.4 | 0.8 |
| ltu-miRn115 | UGACUAUAGCUUCGCAGUGACAA | 23 | 5 | CK758561 | + | 5′ | 182 | −74 | 56.9 | 0.7 |
| ltu-miRn116 | AGGAGUCAUUUUAGAGGAAGGAAU | 24 | 5 | DU169385 | + | 5′ | 112 | −43.9 | 49.2 | 0.8 |
| ltu-miRn117 | AAAACAGAUGAACGGCAUGGAUAU | 24 | 5 | DT595257 | + | 5′ | 143 | −66.3 | 51 | 0.9 |
| ltu-miRn118 | CACUGUUUCCUGUGGUGUGGUCCA | 24 | 5 | FD493303 | + | 5′ | 125 | −30 | 38.2 | 0.6 |
|  |  | 24 | 5 | FD499168 | − | 5′ | 204 | −70.8 | 49.5 | 0.7 |
| ltu-miRn119 | ACUAAGCCUGAGCUGUCGGCCUAU | 24 | 5 | CV003488 | + | 5′ | 199 | −55.4 | 45.6 | 0.6 |
| ltu-miRn120 | AAAAGAUGACAUGGAGAUCAU | 21 | 5 | FD501285 | + | 5′ | 115 | −27.9 | 33.1 | 0.7 |
| ltu-miRn121 | AAGAUCACUAGUCCGAAGAAUCAG | 24 | 5 | FD489615 | + | 5′ | 71 | −24.5 | 39 | 0.8 |
| ltu-miRn122 | ACUACAUGUGUAAGGUCUUCGAUG | 24 | 5 | CK766177 | + | 5′ | 145 | −35.7 | 44.2 | 0.5 |
| ltu-miRn123 | UUGGUUUCAAGCCCAGUGACUCGU | 24 | 5 | DU169428 | + | 5′ | 54 | −15.3 | 45 | 0.6 |
| ltu-miRn124 | CACUUACUAAAUGGGUUCGGGUCG | 24 | 5 | FD496391 | + | 5′ | 79 | −36.3 | 54.1 | 0.8 |
| ltu-miRn125 | GAAUCUCGCGAUUCCGGCCUGGGC | 24 | 5 | DU169566 | + | 5′ | 120 | −41.7 | 51.7 | 0.7 |
| ltu-miRn126 | AGUGUAAACAAACGCGCAUGAGCU | 24 | 5 | DU169547 | + | 5′ | 118 | −38.2 | 47.6 | 0.6 |
| ltu-miRn127 | AGGCGAAAGUGCCGAAUGGACAUG | 24 | 5 | CO999826 | − | 3′ | 55 | −25.8 | 50.8 | 0.8 |
| ltu-miRn128 | GAAACCGAUGGACGGCGUGGA | 21 | 5 | DU169463 | + | 3′ | 80 | −25.2 | 45.3 | 0.6 |
| ltu-miRn129 | CGAGGUGUAGCGCAGUCUGGUCAGC | 25 | 5 | FD493824 | + | 3′ | 96 | −33.4 | 51 | 0.6 |
| ltu-miRn130 | AAAGGCACUAAGGCAUUCAAGGAU | 24 | 5 | DU169479 | − | 3′ | 90 | −14.931 | 29.2 | 0.5 |
| ltu-miRn131 | AAGAGUUUUAACUCGCCGGAGUCG | 24 | 5 | FD492501 | + | 3′ | 143 | −36.8 | 49 | 0.5 |
| ltu-miRn132 | CAACAAGACGCAGCAUGAUCAA | 22 | 5 | FD501188 | − | 3′ | 98 | −41.6 | 49 | 0.8 |
| ltu-miRn133 | GGUCAUGACCGGUCGGCCUUCACA | 24 | 5 | DU169537 | + | 3′ | 159 | −71.8 | 53.7 | 0.8 |
| ltu-miRn134 | GCUGAGCUGAACCAAUCAGGCGU | 23 | 5 | DT581323 | + | 3′ | 143 | −55.7 | 39.7 | 1 |
|  |  | 23 | 5 | DT580670 | + | 3′ | 158 | −72 | 40 | 1.1 |
| ltu-miRn135 | GGACAUGUCUCCGAUCAAUCGGAC | 24 | 5 | DU169617 | + | 3′ | 98 | −45.7 | 48.1 | 0.9 |
| ltu-miRn136 | AAAGGACUCCCCCAAUGCCGACAA | 24 | 4 | DU169354 | − | 5′ | 71 | −18.4 | 44.2 | 0.5 |
| ltu-miRn137 | AGUUGAGGAUUGACAUGUUUGCAU | 24 | 4 | DU169547 | − | 5′ | 130 | −42.1 | 45.6 | 0.7 |
| ltu-miRn138 | UUAGCCUAAACGCUACGUAGCUCA | 24 | 4 | FD491843 | − | 5′ | 52 | −14.9 | 41.4 | 0.6 |
| ltu-miRn139 | AACCAAGCUGUCAAAUCAUAGUAA | 24 | 4 | CK763735 | + | 5′ | 84 | −26.4 | 47.7 | 0.6 |
| ltu-miRn140 | AUCCACCUGGGGUGCUCUACGGAC | 24 | 4 | FD500325 | + | 5′ | 93 | −30.8 | 52.1 | 0.6 |
| ltu-miRn141 | AAGUGGAUCCUUGAAUGCCUUAGU | 24 | 4 | DU169479 | + | 5′ | 97 | −14.4 | 32 | 0.4 |
| ltu-miRn142 | AUACCUAUUUUCGGAUUUCUUCGA | 24 | 4 | CK765847 | + | 5′ | 176 | −37.3 | 32.4 | 0.6 |
| ltu-miRn143 | UGGACUGUCCAUCAUGUGGGGCCC | 24 | 4 | FD501891 | − | 5′ | 120 | −44.5 | 48.4 | 0.7 |
| ltu-miRn144 | AACUUCCUAGAUGUCAUGAUGA | 22 | 4 | FD493002 | − | 5′ | 105 | −29.2 | 38.5 | 0.7 |
| ltu-miRn145 | AGAGGGAUCUUGCGGCUAGAUCAA | 24 | 4 | CK745828 | + | 5′ | 59 | −20.1 | 43.9 | 0.7 |
| ltu-miRn146 | AGCCUGAUCCAAGACUCAAGUGGG | 24 | 4 | DU169538 | + | 5′ | 177 | −39.4 | 35 | 0.6 |
| ltu-miRn147 | AAUGAGGAUGUUGAGAUGGAUGAG | 24 | 4 | FD488051 | − | 5′ | 90 | −21 | 38.5 | 0.6 |
| ltu-miRn148 | AGCAUUGUCAUACUACAACUAGAA | 24 | 4 | DU169525 | + | 5′ | 103 | −23.2 | 39.4 | 0.5 |
| ltu-miRn149 | CACAGGAAACAGUGGUGAUUG | 21 | 4 | FD499168 | + | 5′ | 151 | −87.7 | 42.9 | 1.3 |
| ltu-miRn150 | CUUCAUUUUUUGGACCAUGCCCU | 23 | 4 | FD499611 | + | 5′ | 133 | −28.6 | 43.2 | 0.5 |
| ltu-miRn151 | CGGUCAUGACCGGUCGGAC | 19 | 4 | DU169660 | + | 5′ | 114 | −75.6 | 60.8 | 0.8 |
|  |  | 19 | 4 | DU169650 | + | 5′ | 126 | −72.9 | 60.5 | 0.9 |
|  |  | 19 | 4 | DU169650 | + | 5′ | 126 | −72.9 | 60.5 | 0.9 |
|  |  | 19 | 4 | FD493327 | − | 5′ | 58 | −38.7 | 70 | 0.9 |
| ltu-miRn152 | UAAGAGGUGAAGCUUGUAGUGGAU | 24 | 4 | FD488235 | + | 3′ | 94 | −40.5 | 39 | 1 |
| ltu-miRn153 | AGAUUAGAAAAGAAAUGGGGGAAG | 24 | 4 | CK765847 | + | 3′ | 65 | −13.7 | 32.4 | 0.6 |
| ltu-miRn154 | AGGUAUAUCAACGAAGGAAGCGAG | 24 | 4 | FD498384 | + | 3′ | 108 | −51.5 | 48.2 | 0.9 |
| ltu-miRn155 | AUGGGCGUCUUAGAUUUAUUUCAC | 24 | 4 | DU169412 | + | 3′ | 99 | −40.6 | 40 | 1 |
| ltu-miRn156 | AUCAGAGCUUAGAGUUUGCAUGGU | 24 | 4 | DT600839 | − | 3′ | 58 | −15.9 | 35.9 | 0.7 |
| ltu-miRn157 | UAUGCUAAGGGGCAUGAUCCGGAC | 24 | 4 | DT584024 | − | 3′ | 170 | −51.1 | 45.5 | 0.6 |
| ltu-miRn158 | GAUGGACGGUUGGGAUGAUGGCG | 23 | 4 | CK765557 | + | 3′ | 55 | −21.8 | 60.7 | 0.6 |
| ltu-miRn159 | AUCUAAAAGCACUUGUUGGGGCCC | 24 | 4 | FD492626 | + | 3′ | 118 | −33.5 | 45.8 | 0.6 |
| ltu-miRn160 | CGAGACCCCUUGUUGAAACUCCGC | 24 | 4 | FD498151 | − | 3′ | 55 | −21.1 | 51.8 | 0.7 |
| ltu-miRn161 | GAUCGGAUUGUUCGGCUGCUUGAG | 24 | 4 | FD493303 | − | 3′ | 73 | −23.8 | 54.1 | 0.6 |
| ltu-miRn162 | AAUAAUAGAAACAUUCGUAGACAA | 24 | 4 | FD501505 | + | 3′ | 54 | −11.2 | 30 | 0.6 |
| ltu-miRn163 | AGAAAGUGGUUGUUUGACAUGGUA | 24 | 4 | CK753730 | + | 3′ | 97 | −18.6 | 38 | 0.5 |
| ltu-miRn164 | AUGGAAGCAGUUCUACCAGUCGAG | 24 | 4 | DU169453 | − | 3′ | 174 | −58.8 | 49.4 | 0.7 |
| ltu-miRn165 | CGGUCAUGACCGGUCGGCAGU | 21 | 4 | DU169586 | − | 3′ | 131 | −100.2 | 63.5 | 1.2 |
| ltu-miRn166 | UCAUGGAUGUUUAGAAGAAUUGCU | 24 | 4 | DU169631 | + | 3′ | 55 | −18.7 | 32.2 | 1 |
| ltu-miRn167 | ACAGGAAUUCACGUUCGUUCUGAA | 24 | 4 | DU169354 | + | 3′ | 103 | −23.1 | 39.4 | 0.5 |
| ltu-miRn168 | AUACAUAAAUCUCUGUGGGAACGA | 24 | 4 | DT583018 | − | 3′ | 259 | −54.2 | 33.6 | 0.6 |
| ltu-miRn169 | AGAAUCGAAGAAGCAUCUGA | 20 | 4 | NC_008326 | + | 3′ | 102 | −33.5 | 53.7 | 0.6 |
| ltu-miRn170 | CAAGUGUAAGCUUUCAUCUGA | 21 | 4 | FD496059 | + | 3′ | 82 | −42.2 | 36.4 | 1.3 |
|  |  | 21 | 4 | FD494968 | − | 3′ | 82 | −42.2 | 36.4 | 1.3 |
| ltu-miRn171 | AAGUGGAAAAAGUAACUUAUUUGG | 24 | 4 | DT600974 | + | 3′ | 51 | −22.9 | 29.8 | 1.3 |
| ltu-miRn172 | AGACAAAUGACCUGGCAGCUGACA | 24 | 4 | FD501541 | + | 3′ | 66 | −27.9 | 42.1 | 0.9 |
| ltu-miRn173 | AUUCGUUGAUAGUUGGAAUAGAU | 23 | 4 | NC_008326 | + | 3′ | 118 | −23 | 40.3 | 0.5 |
|  |  | 23 | 4 | FD497914 | − | 3′ | 118 | −23 | 40.3 | 0.5 |
|  |  | 23 | 4 | CK763575 | − | 3′ | 118 | −23 | 40.3 | 0.5 |
| ltu-miRn174 | CACCCUGCUGUCGACGCCA | 19 | 4 | FD499536 | − | 3′ | 135 | −50.8 | 49.6 | 0.7 |
| ltu-miRn175 | CAGAAUGUCUGGAAGCCACCGGUA | 24 | 4 | CV002350 | − | 3′ | 77 | −33.4 | 57 | 0.7 |
| ltu-miRn176 | CCUGAGAUUUGGAUCUGCCUC | 21 | 4 | FD493273 | − | 3′ | 142 | −47.7 | 45.5 | 0.7 |
| ltu-miRn177 | CAAAAUGGAUGGACGGUGUGG | 21 | 3 | CK758537 | + | 5′ | 131 | −37.6 | 46 | 0.6 |
| ltu-miRn178 | ACGGAUUUCAUCUUCGUGUUGAGA | 24 | 3 | FD495813 | + | 5′ | 113 | −21.9 | 33.6 | 0.5 |
| ltu-miRn179 | CUAUCGAGAUUGGUCAGAUAUGCU | 24 | 3 | DU169453 | − | 5′ | 150 | −49 | 49.4 | 0.6 |
| ltu-miRn180 | CAGAACCAUACUGAUGCAUCA | 21 | 3 | FD488540 | + | 5′ | 171 | −57.9 | 47.5 | 0.7 |
| ltu-miRn181 | GUUUGGUUCAAGUGCGAGUGGGAG | 24 | 3 | FD492165 | − | 5′ | 109 | −27.2 | 53.6 | 0.5 |
| ltu-miRn182 | CCUCCUUGUGGGAUCCCAGGA | 21 | 3 | CK760906 | + | 5′ | 71 | −32.9 | 55.8 | 0.8 |
| ltu-miRn183 | AGCUCUGAAUUGUGCAUAUUCUAA | 24 | 3 | DU169575 | + | 5′ | 129 | −32.4 | 34.8 | 0.7 |
| ltu-miRn184 | AACAAUGCUUUGAUGAUGAUGAUG | 24 | 3 | FD489687 | + | 5′ | 91 | −38.6 | 30.9 | 1.3 |
| ltu-miRn185 | AGCUGAAAUCUCGAUGGACCGGCC | 24 | 3 | FD495871 | + | 5′ | 148 | −35.7 | 44.1 | 0.5 |
| ltu-miRn186 | UGAGGAUAUCCUAGAUCUAGCAUA | 24 | 3 | FD496175 | + | 5′ | 99 | −31.3 | 40 | 0.7 |
| ltu-miRn187 | AUCUUGGCUCUGAUAUCAAGUAGA | 24 | 3 | CK744931 | + | 5′ | 117 | −39.9 | 45.5 | 0.7 |
| ltu-miRn188 | AGUGGAUUGGUGUCGCUUUGUGCC | 24 | 3 | DU169613 | + | 5′ | 130 | −32.4 | 40.4 | 0.6 |
| ltu-miRn189 | GGUUUACAGACUGAAGGAGGAGCA | 24 | 3 | DU169460 | + | 5′ | 217 | −67.3 | 43.5 | 0.7 |
| ltu-miRn190 | UACUUAAUUCGAAGGAAGAGAU | 22 | 3 | FD495154 | + | 5′ | 142 | −19.5 | 27 | 0.5 |
|  |  | 22 | 3 | FD487843 | + | 5′ | 142 | −19.5 | 27 | 0.5 |
| ltu-miRn191 | CGAGCUUGCUAAAAAUCCGGUG | 22 | 3 | CK746930 | + | 5′ | 51 | −17.9 | 47.4 | 0.7 |
| ltu-miRn192 | AGAGAGAAGAGAGAGAGAAC | 20 | 3 | FD498437 | + | 5′ | 105 | −38.1 | 52.3 | 0.7 |
| ltu-miRn193 | AAUAGACUUUGAAUAUACUUGCAU | 24 | 3 | FD496686 | + | 5′ | 126 | −28.5 | 28.8 | 0.8 |
| ltu-miRn194 | AGGUCCCUAGAUCCGUACAAUGAA | 24 | 3 | CK745828 | − | 5′ | 72 | −36.3 | 51.3 | 0.9 |
| ltu-miRn195 | AUCACGAUGAUGAUGUGAUCGGAC | 24 | 3 | CK766177 | − | 5′ | 114 | −38.9 | 48.3 | 0.7 |
| ltu-miRn196 | GGCUUUCCAGCUGACGUUGUGGAU | 24 | 3 | DU169637 | − | 5′ | 228 | −52.4 | 39.8 | 0.6 |
| ltu-miRn197 | AUGUAAGACAACCAAGGACAUGUG | 24 | 3 | FD500473 | + | 5′ | 87 | −23.5 | 44.7 | 0.6 |
| ltu-miRn198 | UUCCACAACAUCAUUUCCAGC | 21 | 3 | CK751931 | − | 5′ | 232 | −37 | 37.4 | 0.4 |
| ltu-miRn199 | GUCUCCGAUCAAUCGGACUGGGCU | 24 | 3 | DU169625 | − | 5′ | 63 | −34.8 | 49.5 | 0.8 |
| ltu-miRn200 | AGGGAUGUCGAAAAACGGUAGGUU | 24 | 3 | FD495813 | − | 5′ | 74 | −13.3 | 43.4 | 0.4 |
| ltu-miRn201 | AGGUGAGAGAAAGAGCUUGAAGAA | 24 | 3 | DU169455 | + | 5′ | 60 | −20.6 | 39.4 | 0.8 |
| ltu-miRn202 | AAGUUCGGGCGCUCGGUCGGGCGC | 24 | 3 | DT583603 | − | 5′ | 155 | −87.9 | 64.6 | 0.9 |
| ltu-miRn203 | AAGAACUCAACAGGACCUUACCCC | 24 | 3 | NC_008326 | − | 5′ | 70 | −55.8 | 43.4 | 1.7 |
| ltu-miRn204 | GACAGAUUUUCGAUCAAUCGGACA | 24 | 3 | DU169594 | − | 5′ | 93 | −38.1 | 48.9 | 0.8 |
| ltu-miRn205 | UAUUGGGUGCUUGGGUUAUAACCA | 24 | 3 | CK765274 | − | 5′ | 117 | −32.8 | 48.8 | 0.5 |
| ltu-miRn206 | CAAGCAACUUACACAUGUCUUCCC | 24 | 3 | CK766177 | + | 5′ | 75 | −19 | 46.9 | 0.5 |
| ltu-miRn207 | CGGUCAUGACCGGUCGGCCUU | 21 | 3 | DU169606 | + | 5′ | 142 | −76.4 | 57.4 | 0.9 |
| ltu-miRn208 | UCUUCAUUAAGCUCAACCGACACU | 24 | 3 | DU169395 | − | 5′ | 155 | −40.7 | 36.6 | 0.7 |
| ltu-miRn209 | CAAAAGUUUUGGAUCAAGCUGA | 22 | 3 | DU169463 | + | 5′ | 127 | −27.8 | 31.6 | 0.7 |
| ltu-miRn210 | CUUGCAAUUGGGUCGUUGCGAU | 22 | 3 | NC_008326 | + | 5′ | 57 | −20.2 | 52.4 | 0.6 |
|  |  | 22 | 3 | NC_008326 | − | 5′ | 57 | −20.2 | 52.4 | 0.6 |
|  |  | 22 | 3 | FD491638 | − | 5′ | 57 | −20.2 | 52.4 | 0.6 |
| ltu-miRn211 | GGAGUAUUGUUAGAAGGUGGAUGA | 24 | 3 | FD488870 | − | 5′ | 67 | −16.1 | 31.9 | 0.7 |
|  |  | 24 | 3 | FD488868 | − | 5′ | 67 | −16.1 | 31.9 | 0.7 |
| ltu-miRn212 | UAGAGCUUAGAGUAGGGAAUCAUU | 24 | 3 | CK754156 | − | 5′ | 124 | −32.9 | 41.5 | 0.6 |
| ltu-miRn213 | AAAAUGGAUGGACGGCGUGGAUAA | 24 | 3 | CK759906 | + | 3′ | 135 | −42.7 | 40.4 | 0.8 |
| ltu-miRn214 | UUGAUGUAUGUAUUCUAUAUC | 21 | 3 | DU169645 | − | 3′ | 215 | −85.1 | 43.6 | 0.9 |
| ltu-miRn215 | CACUGUUUCUUGUGGUGUGGUCCA | 24 | 3 | DU169638 | − | 3′ | 260 | −123.1 | 39.5 | 1.2 |
| ltu-miRn216 | CAUGCUCGAGUGGUUGGCUGCAGA | 24 | 3 | FD488523 | + | 3′ | 116 | −59.4 | 65.5 | 0.8 |
| ltu-miRn217 | GCUAGUCUUGUGAUUGGACCCACU | 24 | 3 | FD494761 | + | 3′ | 73 | −20.7 | 38 | 0.7 |
| ltu-miRn218 | GGAGGACUGUCCGAUUGAUCGAAA | 24 | 3 | DU169594 | + | 3′ | 215 | −112.1 | 47.5 | 1.1 |
| ltu-miRn219 | ACCUCCGGACGAGCGUAGCCGAGU | 24 | 3 | DU169417 | + | 3′ | 164 | −62.4 | 50 | 0.7 |
| ltu-miRn220 | GAAGAGGCUGAUGCUCCAUAGCUA | 24 | 3 | DT596828 | + | 3′ | 84 | −32.3 | 52.2 | 0.7 |
| ltu-miRn221 | AAUACACGAAGGAAUCUGUGGCCC | 24 | 3 | FD491688 | + | 3′ | 75 | −23 | 46.3 | 0.6 |
| ltu-miRn222 | CAGGGAGCCCACCGAGACAUGC | 22 | 3 | FD488523 | + | 3′ | 81 | −42 | 63.2 | 0.8 |
| ltu-miRn223 | ACGAAAAUAGACAGAAAUUAAAAA | 24 | 3 | FD490728 | + | 3′ | 51 | −7.4 | 14 | 0.9 |
| ltu-miRn224 | UCAAACAAUGUGGAAGAUCAAAAG | 24 | 3 | FD497350 | + | 3′ | 75 | −25.6 | 37 | 0.9 |
| ltu-miRn225 | CAAUUAGUUGGGAUAAGGCUU | 21 | 3 | FD490991 | + | 3′ | 75 | −19.5 | 37 | 0.7 |
|  |  | 21 | 3 | FD490990 | + | 3′ | 75 | −19.5 | 37 | 0.7 |
| ltu-miRn226 | AAUCUCAUGCUAGAAGACGACAUU | 24 | 3 | DU169613 | − | 3′ | 63 | −11.1 | 37.7 | 0.4 |
| ltu-miRn227 | AGACUUAAGUGGGCUGCACUAAAG | 24 | 3 | DU169508 | − | 3′ | 103 | −29.7 | 40 | 0.7 |
| ltu-miRn228 | UUUCCCAUGAUGGGAUGCUUCACU | 24 | 3 | FD499391 | − | 3′ | 63 | −20.2 | 36.2 | 0.8 |
| ltu-miRn229 | AGGCUAUUAAACCCUUGGGAUCGU | 24 | 3 | FD500678 | + | 3′ | 110 | −29.4 | 44.8 | 0.6 |
| ltu-miRn230 | CUUCAAGGUGCGAGGAAAGGUAAG | 24 | 3 | DU169365 | − | 3′ | 117 | −37.4 | 43.1 | 0.7 |
| ltu-miRn231 | AAAUGAUCUUGCAAGAUGAAUGGA | 24 | 3 | CK763735 | − | 3′ | 59 | −16.2 | 34.8 | 0.7 |
| ltu-miRn232 | ACAUCACGUGUGAUGAGUCC | 20 | 3 | FD492730 | + | 3′ | 72 | −28.1 | 44.7 | 0.8 |
| ltu-miRn233 | AAAUUCAUGAAGAUCCUGCUCAAA | 24 | 3 | DU169546 | − | 3′ | 59 | −19.6 | 40 | 0.8 |
| ltu-miRn234 | AAACCAGACCCAGACCCGUUUAGC | 24 | 3 | FD496391 | + | 3′ | 79 | −36.3 | 54.1 | 0.8 |
| ltu-miRn235 | AAAAUGGAUGGACGGCGUGGA | 21 | 3 | DU169489 | − | 3′ | 235 | −57.7 | 36.5 | 0.7 |
| ltu-miRn236 | AUGCGAGCACUCUUGAUAUGAGCA | 24 | 3 | FD495512 | + | 3′ | 50 | −20 | 51.8 | 0.7 |
| ltu-miRn237 | UUUUGAACAGAACUUCGAUAGGCG | 24 | 3 | DT596920 | + | 3′ | 58 | −13.9 | 43.3 | 0.5 |
| ltu-miRn238 | AGUGGUUGUUUGACAUGGUAUCAG | 24 | 3 | FD491377 | − | 3′ | 82 | −53.2 | 40.9 | 1.5 |
| ltu-miRn239 | AAGUAGAUCCAAAUCUUAGGUGGA | 24 | 3 | FD499559 | − | 3′ | 71 | −18.5 | 35.1 | 0.7 |
| ltu-miRn240 | CGAGUGCACAGCCGGGACUAGGGU | 24 | 3 | DU169428 | + | 3′ | 62 | −20.8 | 57.8 | 0.6 |
| ltu-miRn241 | AUUCAACUUUUGGCUUUUGAAGAG | 24 | 3 | FD499301 | − | 3′ | 60 | −20.7 | 39.4 | 0.8 |
| ltu-miRn242 | AUGAGCAUGCAUCAUAUUUAGGGC | 24 | 3 | FD497230 | + | 3′ | 95 | −18 | 30.7 | 0.6 |
|  |  | 24 | 3 | FD494027 | − | 3′ | 176 | −51.4 | 37.9 | 0.7 |
| ltu-miRn243 | AUGGCCCACUUUCAUCACACCAUC | 24 | 3 | CK756172 | − | 3′ | 99 | −27.8 | 46.1 | 0.6 |
| ltu-miRn244 | UCGGCUCGAUACGAUACGCGAUAU | 24 | 3 | FD487490 | + | 3′ | 70 | −46.9 | 50.7 | 1.3 |
| ltu-miRn245 | AUCCACCAUAAUCUUCGAGACGAU | 24 | 3 | DU169656 | + | 3′ | 116 | −38.5 | 42.1 | 0.7 |
| ltu-miRn246 | ACUUGGUGAGGGCUUUAAUGAUGG | 24 | 3 | DT583481 | − | 3′ | 133 | −55.3 | 51.1 | 0.8 |
| ltu-miRn247 | UUGCAGAAGAGAGAGAUCAC | 20 | 3 | FD491840 | + | 3′ | 102 | −25.3 | 40.7 | 0.6 |
|  |  | 20 | 3 | FD490968 | + | 3′ | 103 | −24.8 | 39.4 | 0.6 |
|  |  | 20 | 3 | CK749047 | + | 3′ | 102 | −27.4 | 41.7 | 0.6 |
| ltu-miRn248 | AAGAUUCUGAUCGAGAAAUGACUA | 24 | 3 | FD494430 | − | 3′ | 64 | −14.9 | 45.7 | 0.5 |
| ltu-miRn249 | ACGACUUCGGGCGGGACCUCGGGC | 24 | 3 | CK746043 | + | 3′ | 83 | −57.9 | 69.8 | 1 |
| ltu-miRn250 | UUUGGAUCAAGCUGAUAUUUGUGU | 24 | 3 | DU169645 | + | 3′ | 139 | −31.6 | 33.1 | 0.7 |
| ltu-miRn251 | UAGAGUAGAAGAUUAUGGAAUAAG | 24 | 3 | FD490811 | + | 3′ | 72 | −13.8 | 23.6 | 0.8 |
| ltu-miRn252 | CUAAAUGGGUUGGGUCCGGGUACC | 24 | 3 | FD493790 | + | 3′ | 115 | −52.4 | 51.2 | 0.8 |
| ltu-miRn253 | AGCUCAAGACUUGAAGAUUAUGCU | 24 | 3 | FD501432 | − | 3′ | 262 | −66.9 | 38.5 | 0.7 |
| ltu-miRn254 | ACAGGACUUGAGGAUGGUUAUGAU | 24 | 3 | DU169647 | + | 3′ | 50 | −19.9 | 39.3 | 0.9 |
|  |  | 24 | 3 | DU169387 | − | 3′ | 69 | −29.9 | 40 | 1 |
| ltu-miRn255 | GCUAGGACACGAGUAGAGGCU | 21 | 3 | DT585602 | + | 3′ | 52 | −16.1 | 50 | 0.6 |
| ltu-miRn256 | AAUAAGUAGAUAAGUAUGUUUGGU | 24 | 3 | DU169512 | − | 3′ | 135 | −33.5 | 30.5 | 0.8 |
| ltu-miRn257 | CCCGUUCAUUGGUUGGAUCUGGCC | 24 | 3 | CK745828 | − | 3′ | 72 | −36.3 | 51.3 | 0.9 |
| ltu-miRn258 | AUUCGUAGAGAUGAUGUGCAAUGG | 24 | 3 | CV005737 | + | 3′ | 96 | −33 | 43.1 | 0.8 |
| ltu-miRn259 | AAAGGGUUUAUAGGUAAAAACAUU | 24 | 3 | FD494232 | + | 3′ | 181 | −71.7 | 31.7 | 1.2 |
| ltu-miRn260 | UGGAUGAAGGGAAAACACAAA | 21 | 3 | DT581072 | + | 3′ | 71 | −17.8 | 39 | 0.6 |
| ltu-miRn261 | CUGGUGCUGUAUCGGAGGAGACGU | 24 | 3 | DT580488 | − | 3′ | 119 | −47.3 | 53.7 | 0.7 |
| ltu-miRn262 | UUGGCAUAGUUCUUGAUGGCC | 21 | 3 | FD493733 | + | 3′ | 84 | −28.5 | 50 | 0.6 |
| ltu-miRn263 | AUAUCGCACGAUAUAUCGUCAAUA | 24 | 3 | CK749760 | − | 3′ | 61 | −34.4 | 36.2 | 1.4 |
| ltu-miRn264 | ACAUUGGAGUUAAUUGCAGAA | 21 | 3 | FD500253 | + | 3′ | 88 | −20.8 | 35.1 | 0.6 |
| ltu-miRn265 | AGGCAUACUUGUAGUCAUCUUAGA | 24 | 3 | FD490143 | − | 3′ | 249 | −47.9 | 38.3 | 0.5 |
| ltu-miRn266 | ACUUUUAAUGUGGAAGAUCUAG | 22 | 3 | CV004423 | + | 3′ | 69 | −20.1 | 45.2 | 0.6 |
|  |  | 22 | 3 | CV003730 | + | 3′ | 69 | −20.1 | 45.2 | 0.6 |
| ltu-miRn267 | AGGCCAGCGGAUCAUAAAAAUCAG | 24 | 3 | DT595285 | + | 3′ | 59 | −20.7 | 48.4 | 0.7 |
| ltu-miRn268 | CAGCAGUAGAUAAGUAACUUA | 21 | 3 | FD491464 | + | 3′ | 50 | −16.8 | 26.8 | 1.1 |
| ltu-miRn269 | GUGUGACUAACAGUGUGGGUGCGU | 24 | 3 | FD490098 | + | 3′ | 142 | −56.4 | 47.3 | 0.8 |
| ltu-miRn270 | CAUUCGAACCAAUCCAAGCCGAGC | 24 | 3 | FD494425 | + | 3′ | 69 | −27.2 | 41.3 | 0.9 |
| ltu-miRn271 | CGAUACGCAUUGUCCGAACAC | 21 | 3 | FD489963 | − | 3′ | 205 | −26.1 | 30.8 | 0.4 |
| ltu-miRn272 | GGACCUUUUUUCGAUCAAUCGGAG | 24 | 3 | DU169594 | − | 3′ | 144 | −64.6 | 47.3 | 0.9 |
| ltu-miRn273 | CAAGCAUUUCAACCCUGUCUG | 21 | 3 | FD496447 | + | 3′ | 103 | −21.6 | 35.8 | 0.6 |

^a^ Chromosome where the read cluster is located.

^b^ Initial free energy.

^c^ The G+C content of pre-miRNAs.

^d^ The minimal folding free energy index(-dG×100/ pre-miRNA Length /(C+G)%).
