# Supplementary material for: Deep Sequencing and Microarray Hybridization Identify Conserved and Species-Specific MicroRNAs during Somatic Embryogenesis in Hybrid Yellow Poplar
Source: PLoS One. 2012 Aug 29;7(8):e43451. doi: 10.1371/journal.pone.0043451 (PMC3430688; doi:10.1371/journal.pone.0043451)
Supplement: Table S3 — Supplement of conserved miRNAs detected in hybrid yellow poplar by microarray analysis. (DOC) [file pone.0043451.s004.doc]

## Table S3. Supplement of conserved miRNAs detected in hybrid yellow poplar (*L. tulipifera×L. chinense*) by microarray analysis.

| **Reporter name** | **Target sequence (5′ to 3′)** | **Reporter name** | **Target sequence (5' to 3')** |
| --- | --- | --- | --- |
| zma-miR529 | AGAAGAGAGAGAGUACAGCCU | peu-miR2915 | CCCGUCUAGCUCAGUUGGUA |
| zma-miR408b* | CAGGGACGAGGCAGAGCAUGG | peu-miR2914 | CAUGGUGGUGACGGGUGACGGAG |
| zma-miR398b* | GGGGCGGACUGGGAACACAUG | peu-miR2911 | GGCCGGGGGACGGGCUGGGA |
| zma-miR397a | UCAUUGAGCGCAGCGUUGAUG | peu-miR2910 | UAGUUGGUGGAGCGAUUUGUC |
| zma-miR396f* | GGUCAAGAAAGCUGUGGGAAG | osa-miR529b | AGAAGAGAGAGAGUACAGCUU |
| zma-miR396e* | GGUCAAGAAAGCCGUGGGAAG | osa-miR415 | AACAGAACAGAAGCAGAGCAG |
| zma-miR396a* | GUUCAAUAAAGCUGUGGGAAA | osa-miR397b | UUAUUGAGUGCAGCGUUGAUG |
| zma-miR2118f | UUCCCAAUGCCUUCCAUGCCUA | osa-miR319a | UUGGACUGAAGGGUGCUCCC |
| zma-miR171f | UUGAGCCGUGCCAAUAUCACA | osa-miR2118p | UUCCCGAUGCCUCCCAUGCCUA |
| zma-miR171c | UGACUGAGCCGUGCCAAUAUC | osa-miR2118f | UUCCUGAUGCCUCCCAUUCCUA |
| zma-miR171b | UUGAGCCGUGCCAAUAUCAC | osa-miR2118e | UUCCCAAUGCCUCCCAUGCCUA |
| zma-miR166m* | GGAAUGUUGGCUGGCUCGAGG | osa-miR2118d | UUCCUGAUGCCUCCCAUGCCUA |
| zma-miR166c* | GGAAUGUUGUCUGGCUCGAGG | osa-miR2118b | UUCCCGAUGCCUCCCAUUCCUA |
| zma-miR166a* | GGAAUGUUGUCUGGCUCGGGG | osa-miR2118a | UUCUCGAUGCCUCCCAUUCCUA |
| zma-miR162 | UCGAUAAACCUCUGCAUCCA | osa-miR2091-5p | UCAACCGAGCCGAGGAGGAGG |
| zma-miR159e | AUUGGUUUGAAGGGAGCUCCA | osa-miR1881 | AAUGUUAUUGUAGCGUGGUGGUGU |
| zma-miR156a* | GCUCACUUCUCUCUCUGUCAGU | osa-miR1858a | GAGAGGAGGACGGAGUGGGGC |
| vvi-miR482 | UCUUUCCUACUCCUCCCAUUCC | osa-miR1846a-5p | AGUGAGGAGGCCGGGGCCGCU |
| vvi-miR171h | UGGUUGAGCCGCGCCAAUAUC | osa-miR168b | AGGCUUGGUGCAGCUCGGGAA |
| vvi-miR167c | UGAAGCUGCCAGCAUGAUCUC | osa-miR167d | UGAAGCUGCCAGCAUGAUCUG |
| vvi-miR166a | UCGGACCAGGCUUCAUUCC | osa-miR166m | UCGGACCAGGCUUCAUUCCCU |
| vvi-miR156h | UGACAGAAGAGAGAGAGCAU | osa-miR166k | UCGGACCAGGCUUCAAUCCCU |
| vvi-miR156e | UGACAGAGGAGAGUGAGCAC | osa-miR166g | UCGGACCAGGCUUCAUUCCUC |
| tae-miR1134 | CAACAACAACAAGAAGAAGAAGAU | osa-miR159f | CUUGGAUUGAAGGGAGCUCUA |
| tae-miR1125 | AACCAACGAGACCAACUGCGGCGG | osa-miR159e | AUUGGAUUGAAGGGAGCUCCU |
| tae-miR1124 | GCAGGACGUGAAGAGCGAGUCC | osa-miR159d | AUUGGAUUGAAGGGAGCUCCG |
| sof-miR408e | CUGCACUGACUCUUCCCUGGC | osa-miR159c | AUUGGAUUGAAGGGAGCUCCA |
| sof-miR168b | UCGCUUGGGCAGAUCGGGAC | osa-miR159a.1 | UUUGGAUUGAAGGGAGCUCUG |
| sof-miR159e | UUUGGAUUGAAAGGAGCUCUU | osa-miR156l | CGACAGAAGAGAGUGAGCAUA |
| smo-miR156d | UUGACAGAAGACAGGGAGCAC | osa-miR1436 | ACAUUAUGGGACGGAGGGAGU |
| smo-miR156c | UUGACAGAAGAAAGAGAGCAC | mtr-miR171c | UGAUUGAGCCGUGCCAAUAUU |
| smo-miR156b | CUGACAGAAGAUAGAGAGCAC | mtr-miR168 | UUGCUUGGUGCUGGUCGGGAA |
| smo-miR1088-5p | CAGAAGAAAGAGAGCACGCAU | mtr-miR166b | UCGGACCAGGCUUCAUUCCUA |
| smo-miR1088-3p | GCGUGCUCUUUUUCUUCUGUC | gma-miR390b | AAGCUCAGGAGGGAUAGCACC |
| sly-miR397 | AUUGAGUGCAGCGUUGAUGA | gma-miR390a-3p | CGCUAUCCAUCCUGAGUUUC |
| sly-miR319 | CUUGGACUGAAGGGAGCUCC | ghr-miR2950 | UGGUGUGCAGGGGGUGGAAUA |
| sbi-miR398 | UGUGUUCUCAGGUCGCCCCCG | ghr-miR156c | UGUCAGAAGAGAGUGAGCAC |
| sbi-miR166k | UCGGACCAGGCUUCAUUCCU | crt-miR166b | UCGGACCAGGCUUCAUUCCCUU |
| sbi-miR166a | UCGGACCAGGCUUCAUUCCC | crt-miR166a | UCGGACCAGGCUUCAUUCCCGU |
| sbi-miR159b | CUUGGAUUGAAGGGAGCUCCU | cre-miR1160.2 | UGACAAGGAAGCAGAGCGGAU |
| rco-miR171g | AGAUUGAGCCGCGCCAAUAUC | cre-miR1148.1 | CCAACGUGCAGGGGGACAUGG |
| ptc-miR482.2 | UCUUGCCUACUCCUCCCAUU | cre-miR1144b | UGGGUAGUGUGGCGGCAGGCAG |
| ptc-miR474c | CAAAAGCUGUUGGGUUUGGCUGGG | ccl-miR167a | UGAAGCUGCCAGCAUGAUCUGA |
| ptc-miR474b | CAAAAGUUGUUGGGUUUGGCUGGG | bna-miR397a | UCAUUGAGUGCAGCGUUGAUGU |
| ptc-miR474a | CAAAAGUUGCUGGGUUUGGCUGGG | bna-miR171g | UGAUUGAGCCGCGCCAAUAUCU |
| ptc-miR472b | UUUUCCCAACUCCACCCAUCCC | bna-miR167a | UGAAGCUGCCAGCAUGAUCUAA |
| ptc-miR397b | CCAUUGAGUGCAGCGUUGAUG | bna-miR156a | UGACAGAAGAGAGUGAGCACA |
| ptc-miR319e | UUGGACUGAAGGGAGCUCCU | bdi-miR156 | UGACAGAAGAGAGAGAGCACA |
| ptc-miR171k | GGAUUGAGCCGCGCCAAUAUC | ath-miR854a | GAUGAGGAUAGGGAGGAGGAG |
| ptc-miR167h | UGAAGCUGCCAACAUGAUCUG | ath-miR408 | AUGCACUGCCUCUUCCCUGGC |
| ptc-miR166p | UCGGACCAGGCUCCAUUCCUU | ath-miR398a | UGUGUUCUCAGGUCACCCCUU |
| ptc-miR166n | UCGGACCAGGCUUCAUUCCUU | ath-miR395b | CUGAAGUGUUUGGGGGGACUC |
| ptc-miR159f | AUUGGAGUGAAGGGAGCUCGA | ath-miR319c | UUGGACUGAAGGGAGCUCCUU |
| ptc-miR1450 | UUCAAUGGCUCGGUCAGGUUAC | ath-miR2936 | CUUGAGAGAGAGAACACAGACG |
| ptc-miR1447 | CAGAAUUGCAGUGCCUUGAUU | ath-miR171b | UUGAGCCGUGCCAAUAUCACG |
| pta-miR948 | UCAGGCUGUGUGGGAUCCGG | ath-miR171a | UGAUUGAGCCGCGCCAAUAUC |
| pta-miR319 | UUGGACUGAAGGGAGCUCC | ath-miR167d | UGAAGCUGCCAGCAUGAUCUGG |
| pta-miR159c | CUUGGAUUGAAGGGAGCUCCC | ath-miR167c | UAAGCUGCCAGCAUGAUCUUG |
| pta-miR159a | UUGGAUUGAAGGGAGCUCCA | ath-miR162a | UCGAUAAACCUCUGCAUCCAG |
| pta-miR156b | CAGAAGAUAGAGAGCACAAC | ath-miR159c | UUUGGAUUGAAGGGAGCUCCU |
| pta-miR156a | CAGAAGAUAGAGAGCACAUC | ath-miR159b | UUUGGAUUGAAGGGAGCUCUU |
| ppt-miR896 | GUCAAUUUGGCCGAGUGGUUAAGGC | ath-miR159a | UUUGGAUUGAAGGGAGCUCUA |
| ppt-miR894 | CGUUUCACGUCGGGUUCACC | ath-miR156h | UGACAGAAGAAAGAGAGCAC |
| ppt-miR529g | CGAAGAGAGAGAGCACAGUCC | ath-miR156g | CGACAGAAGAGAGUGAGCAC |
| ppt-miR529e | AGAAGAGAGAGAGUACAGCCC | aqc-miR535 | UGACAACGAGAGAGAGCACGCG |
| ppt-miR529a | CGAAGAGAGAGAGCACAGCCC | aqc-miR529 | AGAAGAGAGAGAGCACAACCC |
| ppt-miR395 | CUGAAGCGUUUGGGGGAAGG | aqc-miR482a | UCUUGCCGACUCCUCCCAUACC |
| ppt-miR390c | GAGCUCAGGAGGGAUAGCGCC | aqc-miR171f | UAAUUGAGCCGUGCCAAUAUC |
| ppt-miR319c | CUUGGACUGAAGGGAGCUCCC | aqc-miR167 | UCAAGCUGCCAGCAUGAUCUA |
| ppt-miR171b | UUGAGCCGCGCCAAUAUCACA | aqc-miR159 | UUUGGACUGAAGGGAGCUCUA |
| ppt-miR171a | UGAGCCGCGCCAAUAUCACAU | ahy-miR408-5p | CUGGGAACAGGCAGAGCAUGA |
| ppt-miR167 | GGAAGCUGCCAGCAUGAUCCU | ahy-miR398 | UGUGUUCUCAGGUCACCCCU |
| ppt-miR166j | UCCGGACCAGGCUUCAUUCCC | ahy-miR3509-3p | AUCUAACGACUCUCAGAUAUCA |
| ppt-miR1066 | ACAUGUUGCAGAGCGGGGUAC | ahy-miR156c | UUGACAGAAGAGAGAGAGCAC |
| peu-miR2916 | UGGGGACUCGAAGACGAUCAUAU |  |  |

^*^The mature miRNA got from a star stand which is complementary to the guide strand.

Abbreviations: zma, *Zea mays*; vvi, *Vitis vinifera*; tae, *Triticum aestivum*; sof, *Saccharum officinarum*; smo, *Selaginella moellendorffii*; sly, *Solanum lycopersicum*; sbi, *Sorghum bicolor*; rco, *Ricinus communis*; ptc, *Populus trichocarpa*; pta, *Pinus taeda*; ppt, *Physcomitrella patens*; peu, *Populus euphratica*; osa, *Oryza sativa*; mtr, *Medicago truncatula*; gma, *Glycine max*; ghr, *Gossypium hirsutum*; crt, *Citrus reticulate*; cre, *Chlamydomonas reinhardtii*; ccl, *Citrus Clementine*; bna, *Brassica napus*; bdi, *Brachypodium distachyon*; ath, *Arabidopsis thaliana*; aqc, *Aquilegia caerulea*; ahy, *Arachis hypogaea*.
