# Supplementary material for: Deep Sequencing and Microarray Hybridization Identify Conserved and Species-Specific MicroRNAs during Somatic Embryogenesis in Hybrid Yellow Poplar
Source: PLoS One. 2012 Aug 29;7(8):e43451. doi: 10.1371/journal.pone.0043451 (PMC3430688; doi:10.1371/journal.pone.0043451)
Supplement: Table S4 — Target proposed function of conserved miRNAs in hybrid yellow poplar. (DOC) [file pone.0043451.s005.doc]

## Table S4. Target proposed function of conserved miRNAs in hybrid yellow poplar (*L. tulipifera × L. chinense*).

| **miRNA name** | **Target accession** | **Expectation^a^** | **UPE^b^** | **Target description** |
| --- | --- | --- | --- | --- |
| ltu-MIR156a | AT3G15270.1 | 1 | 8.059 | SPL5 (SQUAMOSA PROMOTER BINDING PROTEIN-LIKE 5); DNA binding / transcription factor |
| ltu-MIR156c | AT2G33810.1 | 1 | 15.985 | SPL3 (SQUAMOSA PROMOTER BINDING PROTEIN-LIKE 3); DNA binding / transcription factor |
| ltu-MIR156d | AT3G15270.1 | 0 | 8.059 | SPL5; DNA binding / transcription factor |
| ltu-MIR156e | AT2G33810.1 | 1.5 | 15.985 | SPL3; DNA binding / transcription factor |
| ltu-MIR156g | AT1G27370.1 | 0.5 | 12.296 | SPL10 (SQUAMOSA PROMOTER BINDING PROTEIN-LIKE 10) |
| ltu-MIR156h | AT3G28690.1 | 1 | 11.227 | Protein kinase, putative |
| ltu-MIR156i | AT2G47590.1 | 0.5 | 3.553 | PHR2 (Photolyase/blue-light receptor 2); DNA photolyase |
| ltu-MIR156j | AT1G69170.1 | 0 | 16.454 | SPL6 (SQUAMOSA PROMOTER BINDING PROTEIN-LIKE 6) |
| ltu-MIR156l | AT4G08500.1 | 0 | 1.158 | MEKK1, ATMEKK1, MAPKKK8, ARAKIN | MEKK1 (MAP KINASE KINASE KINASE 1); DNA binding / MAP kinase kinase kinase1/ kinase/ kinase binding / protein binding |
| ltu-MIR157b | AT1G27360.2 | 1 | 10.981 | SPL11 (SQUAMOSA PROMOTER BINDING PROTEIN-LIKE 11) |
| ltu-MIR159d | AT4G09650.1 | 1.5 | 2.432 | ATPD (ATP SYNTHASE DELTA-SUBUNIT GENE); hydrogen ion transporting ATP synthase, rotational mechanism / proton-transporting ATPase, rotational mechanism |
| ltu-MIR160d | AT2G28350.1 | 0 | 18.139 | ARF10 (AUXIN RESPONSE FACTOR 10); miRNA binding / transcription factor |
| ltu-MIR162a | AT2G23180.1 | 1 | 19.129 | CYP96A1; electron carrier/ heme binding / iron ion binding / monooxygenase / oxygen binding |
| ltu-MIR164e | AT5G61430.1 | 0 | 11.289 | ANAC100 (ARABIDOPSIS NAC DOMAIN CONTAINING PROTEIN 100); transcription factor |
| ltu-MIR165b | AT2G34710.1 | 0.5 | 21.099 | PHB, ATHB14, ATHB-14, PHB-1D | PHB (PHABULOSA); DNA binding / transcription factor |
| ltu-MIR166d | AT2G34710.1 | 1.5 | 21.099 | PHB, ATHB14, ATHB-14, PHB-1D | PHB (PHABULOSA); DNA binding / transcription factor |
| ltu-MIR166e | AT1G12470.1 | 1.5 | 20.101 | Pep3/Vps18/deep orange family protein |
| ltu-MIR166i | AT5G50490.1 | 1.5 | 15.838 | NF-YC5 (NUCLEAR FACTORY, SUBUNIT C5); DNA binding / transcription factor |
| ltu-MIR166k | AT2G34710.1 | 2 | 21.099 | PHB(PHABULOSA), ATHB14, ATHB-14, PHB-1D; DNA binding / transcription factor |
| ltu-MIR166m | AT2G34710.1 | 1 | 21.099 | PHB, ATHB14, ATHB-14, PHB-1D; DNA binding / transcription factor |
| ltu-MIR166q | AT2G34710.1 | 1 | 21.099 | PHB, ATHB14, ATHB-14, PHB-1D; DNA binding / transcription factor |
| ltu-MIR167e | AT5G41300.1 | 2 | 19.68 | Receptor-like protein kinase-related |
| ltu-MIR167f | AT5G41300.1 | 2 | 19.68 | Receptor-like protein kinase-related |
| ltu-MIR167h | AT3G06060.1 | 2 | 15.814 | SDR (short-chain dehydrogenase/reductase ) family protein |
| ltu-MIR168a | AT3G19740.1 | 2 | 18.071 | ATP binding / ATPase/ nucleoside-triphosphatase/ nucleotide binding |
| ltu-MIR168b | AT5G07140.1 | 1.5 | 18.054 | Protein kinase family protein |
| ltu-MIR168e | AT3G21100.1 | 1.5 | 18.44 | RNA binding / nucleic acid binding / nucleotide binding |
| ltu-MIR169s | AT1G17590.2 | 1 | 15.898 | NF-YA8 (NUCLEAR FACTOR Y, SUBUNIT A8); transcription factor |
| ltu-MIR170 | AT3G60630.1 | 1 | 14.202 | Scarecrow transcription factor family protein |
| ltu-MIR171d | AT3G60630.1 | 0 | 14.419 | Scarecrow transcription factor family protein |
| ltu-MIR171d | AT3G60630.1 | 0 | 14.419 | Scarecrow transcription factor family protein |
| ltu-MIR171i | AT3G60630.1 | 0.5 | 14.202 | Scarecrow transcription factor family protein |
| ltu-MIR172e | AT2G39250.1 | 0.5 | 12.263 | SNZ (SCHNARCHZAPFEN); DNA binding / transcription factor |
| ltu-MIR2118p | AT5G48770.1 | 0.5 | 16.458 | Disease resistance protein (TIR-NBS-LRR class), putative |
| ltu-MIR319a | AT2G26950.1 | 1.5 | 18.403 | AtMYB104 (myb domain protein 104); DNA binding / transcription factor |
| ltu-MIR319a | AT2G26950.1 | 1.5 | 18.403 | AtMYB104; DNA binding / transcription factor |
| ltu-MIR3522a | AT3G17750.1 | 0.5 | 15.474 | Protein kinase family protein |
| ltu-MIR390a | AT2G41570.1 | 2.5 | 12.401 | Transposable element gene |
| ltu-MIR390b | AT4G00160.1 | 1.5 | 17.09 | F-box family protein |
| ltu-MIR390d | AT5G57735.1 | 0.5 | 8.646 | TASIR-ARF; other RNA |
| ltu-MIR394a | AT1G27340.1 | 1 | 14.87 | F-box family protein |
| ltu-MIR396 | AT3G52910.1 | 0 | 14.358 | AtGRF4 (GROWTH-REGULATING FACTOR 4); transcription activator |
| ltu-MIR396e | AT2G03250.1 | 2 | 17.486 | EXS family protein / ERD1/XPR1/SYG1 family protein |
| ltu-MIR396g | AT2G04270.4 | 1 | 19.2 | RNEE/G | RNEE/G (RNASE E/G-LIKE); endoribonuclease |
| ltu-MIR397a | AT2G29130.1 | 1.5 | 11.407 | LAC2 (laccase 2), ATLAC2; laccase |
| ltu-MIR397b | AT2G29130.1 | 0.5 | 11.407 | LAC2, ATLAC2; laccase |
| ltu-MIR397c | AT2G29130.1 | 1 | 11.407 | LAC2, ATLAC2; laccase |
| ltu-MIR397d | AT2G29130.1 | 1.5 | 11.407 | LAC2, ATLAC2; laccase |
| ltu-MIR398a | AT1G35770.1 | 2 | 18.675 | Transposable element gene |
| ltu-MIR398b | AT1G35770.1 | 2 | 18.675 | Transposable element gene |
| ltu-MIR399b | AT2G33770.1 | 0 | 19.497 | UBC24, ATUBC24, PHO (PHOSPHATE 2); ubiquitin-protein ligase |
| ltu-MIR408b | AT2G02850.1 | 1 | 23.175 | ARPN (PLANTACYANIN); copper ion binding / electron carrier |
| ltu-MIR472b | AT5G17680.1 | 0.5 | 16.122 | Disease resistance protein (TIR-NBS-LRR class), putative |
| ltu-MIR477 | AT4G02220.1 | 1.5 | 14.773 | Zinc finger (MYND type) family protein / programmed cell death 2 C-terminal domain-containing protein |
| ltu-MIR482a | AT5G05400.1 | 1.5 | 19.44 | Disease resistance protein (CC-NBS-LRR class), putative |
| ltu-MIR528a | AT1G80370.1 | 2 | 19.752 | CYCA2;4 (Cyclin A2;4); cyclin-dependent protein kinase regulator |
| ltu-MIR529 | AT1G27360.1 | 1 | 10.981 | SPL11 |
| ltu-MIR529d | AT1G03730.1 | 0 | 9.379 | Unknown protein REVERSE [PFAM] |
| ltu-MIR535a | AT5G44590.1 | 1.5 | 17.171 | Unknown protein FORWARD [PFAM] |
| ltu-MIR894 | AT5G35390.1 | 2 | 20.44 | Leucine-rich repeat transmembrane protein kinase, putative |
| ltu-MIR1511 | AT3G28160.1 | 0 | 19.193 | Transposable element gene |

^a^The scoring schema to score the complementarity between small RNA (mainly including miRNA and ta-siRNA) and their target transcript. In this study, we set 2.0 as the threshold of maximum expectation.

^b^ Unpaird energy, maximum energy allowed to unpair the target site.
