# Supplementary material for: Deep Sequencing and Microarray Hybridization Identify Conserved and Species-Specific MicroRNAs during Somatic Embryogenesis in Hybrid Yellow Poplar
Source: PLoS One. 2012 Aug 29;7(8):e43451. doi: 10.1371/journal.pone.0043451 (PMC3430688; doi:10.1371/journal.pone.0043451)
Supplement: Table S5 — Target proposed function of species-specific miRNAs in hybrid yellow poplar. (DOC) [file pone.0043451.s006.doc]

## **Table S5. Target proposed function of species-specific miRNAs in hybrid yellow poplar (*L. tulipifera × L. chinense*).**

| **miRNA name** | **Target accession** | **Expectation^a^** | **UPE^b^** | **Target description** |
| --- | --- | --- | --- | --- |
| ltu-miRn1 | AT3G55430.1 | 1.5 | 21.128 | Glycosyl hydrolase family 17 protein /beta-1,3-glucanase, putative |
| ltu-miRn2 | AT5G33624.1 | 2 | 20.549 | Transposable element gene |
| ltu-miRn3 | AT5G17680.1 | 1.5 | 16.122 | Disease resistance protein (TIR-NBS-LRR class), putative |
| ltu-miRn4 | AT1G59710.1 | 1 | 20.649 | Actin cross-linking protein (InterPro:IPR008999) |
| ltu-miRn5 | AT4G24680.1 | 0.5 | 21.862 | BAT2, N-terminal (InterPro:IPR009738) |
| ltu-miRn6 | AT1G10740.1 | 1 | 15.815 | Unknown protein |
| ltu-miRn7 | AT5G15990.1 | 1.5 | 17.323 | Transposable element gene |
| ltu-miRn8 | AT4G29480.1 | 1.5 | 19.752 | Mitochondrial ATP synthase g subunit family protein |
| ltu-miRn9 | AT3G30145.1 | 1.5 | 10.38 | Transposable element gene |
| ltu-miRn10 | AT4G33810.1 | 1 | 23.133 | Lycosyl hydrolase family 10 protein |
| ltu-miRn11 | AT1G48500.3 | 2.5 | 15.49 | JAZ4 (JASMONATE-ZIM-DOMAIN PROTEIN 4), TIFY6A |
| ltu-miRn12 | AT4G19610.1 | 0.5 | 19.541 | RNA binding / nucleic acid binding / nucleotide binding |
| ltu-miRn13 | AT2G20460.1 | 1.5 | 8.466 | Transposable element gene |
| ltu-miRn17 | AT3G09190.1 | 2 | 15.006 | Sugar binding |
| ltu-miRn19 | AT3G15580.1 | 2 | 16.344 | ATG8I, APG8H, APG8 activating enzyme/ APG8-specific protease/ Atg8 ligase / microtubule binding |
| ltu-miRn21 | AT4G18360.2 | 2 | 8.698 | (S)-2-hydroxy-acid oxidase, peroxisomal, putative / glycolate oxidase, putative / short chain alpha-hydroxy acid oxidase, putative |
| ltu-miRn22 | AT4G28710.1 | 2 | 15.288 | XIH, ATXIH, motor |
| ltu-miRn24 | AT5G35160.1 | 0.5 | 14.632 | Contains InterPro DOMAIN(s): Nonaspanin (TM9SF) (InterPro:IPR004240) |
| ltu-miRn25 | AT3G21560.1 | 1.5 | 7.893 | UGT84A2, UDP-glycosyltransferase/ sinapate 1-glucosyltransferase |
| ltu-miRn26 | AT4G16690.1 | 1.5 | 21.618 | ATMES16, MES16 (METHYL ESTERASE 16), catalytic/ hydrolase, acting on ester bonds / methyl indole-3-acetate esterase/ methyl jasmonate esterase |
| ltu-miRn27 | AT3G06000.1 | 1 | 14.078 | Leucine-rich repeat family protein |
| ltu-miRn29 | AT5G03940.1 | 3 | 20.792 | FFC, 54CP, CPSRP54, CPSRP54; 7S RNA binding / GTP binding / mRNA binding / signal sequence binding |
| ltu-miRn30 | AT3G62040.1 | 2.5 | 15.64 | Catalytic/ hydrolase |
| ltu-miRn35 | AT4G08105.1 | 1 | 23.297 | Transposable element gene |
| ltu-miRn36 | AT1G49340.1 | 2.5 | 13.439 | ATPI4K ALPHA, 1-phosphatidylinositol 4-kinase/ inositol or phosphatidylinositol kinase |
| ltu-miRn38 | AT4G07810.1 | 2.5 | 16.529 | Transposable element gene |
| ltu-miRn39 | AT3G01760.1 | 1 | 19.134 | Lysine and histidine specific transporter, putative |
| ltu-miRn40 | AT1G34240.1 | 1 | 14.23 | Transposable element gene |
| ltu-miRn42 | AT3G50430.1 | 1.5 | 15.617 | Unknown protein |
| ltu-miRn43 | AT4G35300.3 | 1.5 | 17.46 | TMT2 (TONOPLAST MONOSACCHARIDE TRANSPORTER2), carbohydrate transmembrane transporter/ nucleoside transmembrane transporter/ sugar |
| ltu-miRn44 | AT4G36970.1 | 1.5 | 20.731 | Remorin family protein |
| ltu-miRn45 | AT1G33980.2 | 1.5 | 13.662 | ATUPF3, Smg-4/UPF3 family protein |
| ltu-miRn46 | AT1G16480.1 | 1.5 | 18.048 | Pentatricopeptide (PPR) repeat-containing protein |
| ltu-miRn48 | AT5G57160.1 | 1.5 | 19.724 | ATLIG4, LIG4, DNA ligase (ATP)/ protein binding |
| ltu-miRn51 | AT3G16560.1 | 0.5 | 1.766 | Protein phosphatase 2C-related / PP2C-related |
| ltu-miRn54 | AT1G22240.1 | 2.5 | 22.297 | APUM8 (Arabidopsis Pumilio 8), RNA binding / binding |
| ltu-miRn55 | AT5G33428.1 | 1.5 | 12.837 | Transposable element gene |
| ltu-miRn56 | AT1G52080.1 | 2 | 4.092 | AR791, actin binding |
| ltu-miRn57 | AT4G14400.1 | 3 | 22.119 | ACD6 (ACCELERATED CELL DEATH 6), protein binding |
| ltu-miRn58 | AT1G47810.1 | 2.5 | 22.84 | F-box family protein |
| ltu-miRn59 | AT2G45170.2 | 1 | 17.717 | AtATG8e, microtubule binding |
| ltu-miRn63 | AT3G58950.1 | 2 | 18.24 | F-box family protein |
| ltu-miRn64 | AT3G25585.1 | 2.5 | 8.777 | AAPT2 (AMINOALCOHOLPHOSPHOTRANSFERASE), ATAAPT2, phosphatidy ltransferase/ phosphotransferase |
| ltu-miRn66 | AT3G43955.1 | 0 | 14.285 | Transposable element gene |
| ltu-miRn67 | AT4G34110.1 | 1.5 | 24.061 | PAB2, PABP2, ATPAB2, RNA binding / translation initiation factor |
| ltu-miRn68 | AT5G13655.1 | 3 | 14.771 | Transposable element gene |
| ltu-miRn69 | AT5G08320.1 | 2.5 | 13.323 | Unknown protein |
| ltu-miRn71 | AT4G20130.1 | 2 | 19.255 | PTAC14 (PLASTID TRANSCRIPTIONALLY ACTIVE14) |
| ltu-miRn72 | AT3G05905.1 | 2.5 | 12.259 | Other RNA |
| ltu-miRn74 | AT4G35130.1 | 1 | 19.882 | PPR repeat-containing protein |
| ltu-miRn75 | AT2G46430.2 | 2 | 14.806 | ATCNGC3(CYCLIC NUCLEOTIDE GATED CHANNEL 3), CNGC3, CNGC3.C, calmodulin binding / cyclic nucleotide binding / ion channel |
| ltu-miRn77 | AT1G65960.1 | 2.5 | 21.36 | GAD2 (GLUTAMATE DECARBOXYLASE 2), calmodulin binding / glutamate decarboxylase |
| ltu-miRn78 | AT5G11260.1 | 2 | 24.095 | HY5 (ELONGATED HYPOCOTYL 5), TED 5, DNA binding / double-stranded DNA binding / transcription factor |
| ltu-miRn80 | AT1G26990.1 | 2.5 | 14.012 | Transposable element gene |
| ltu-miRn81 | AT3G22136.1 | 3 | 18.88 | Transposable element gene |
| ltu-miRn82 | AT4G25970.1 | 1 | 17.941 | PSD3 (phosphatidylserine decarboxylase 3) |
| ltu-miRn83 | AT5G25520.2 | 1.5 | 14.885 | Transcription elongation factor-related |
| ltu-miRn84 | AT2G21720.1 | 3 | 15.03 | Unknown protein |
| ltu-miRn87 | AT5G38600.1 | 1.5 | 13.263 | PSP (proline-rich spliceosome-associated) family protein / zinc knuckle (CCHC-type) family protein |
| ltu-miRn88 | AT5G15280.1 | 1.5 | 15.391 | PPR repeat-containing protein |
| ltu-miRn90 | AT5G23980.1 | 0.5 | 18.999 | ATFRO4, FRO4 (FERRIC REDUCTION OXIDASE 4), ferric-chelate reductase |
| ltu-miRn91 | AT1G29357.1 | 2.5 | 10.739 | Other RNA |
| ltu-miRn93 | AT3G04605.1 | 2.5 | 11.479 | Transposable element gene |
| ltu-miRn94 | AT4G21903.1 | 1.5 | 16.622 | Antiporter/ drug transporter |
| ltu-miRn95 | AT4G06631.1 | 2.5 | 13.834 | Transposable element gene |
| ltu-miRn97 | AT1G14690.2 | 3 | 13.726 | MAP65-7 (MICROTUBULE-ASSOCIATED PROTEIN 65-7) |
| ltu-miRn102 | AT1G72290.1 | 2 | 14.386 | Trypsin and protease inhibitor family protein / Kunitz family protein |
| ltu-miRn105 | AT5G07990.1 | 2.5 | 14.704 | TT7(TRANSPARENT TESTA 7), CYP75B1, D501, flavonoid 3'-monooxygenase/ oxygen binding |
| ltu-miRn106 | AT2G21390.1 | 1 | 12.015 | Coatomer protein complex, subunit alpha, putative |
| ltu-miRn107 | AT3G28660.1 | 2.5 | 14.921 | PPR repeat-containing protein |
| ltu-miRn108 | AT5G46750.1 | 2 | 14.377 | AGD9 (ARF-GAP DOMAIN 9), ARF GTPase activator/ DNA binding / zinc ion binding |
| ltu-miRn109 | AT5G25040.1 | 1.5 | 19.741 | transporter |
| ltu-miRn111 | AT1G75760.1 | 2.5 | 18.443 | ER lumen protein retaining receptor family protein |
| ltu-miRn112 | AT1G08680.1 | 1.5 | 20.13 | AGD14, ZIGA4 (ARF GAP-like zinc finger-containing protein ZiGA4), ARF GTPase activator/ DNA binding / zinc ion binding |
| ltu-miRn113 | AT2G48030.1 | 2 | 17.994 | Endonuclease/exonuclease/phosphatase family protein |
| ltu-miRn114 | AT2G30360.1 | 2 | 18.615 | CIPK11, PKS5, SIP4 (SOS3-INTERACTING PROTEIN 4), SNRK3.22 , kinase/ protein kinase |
| ltu-miRn115 | AT3G11960.2 | 2 | 15.193 | CPSF (cleavage and polyadenylation specificity factor) |
| ltu-miRn116 | AT1G42888.1 | 1.5 | 16.947 | Transposable element gene |
| ltu-miRn117 | AT3G15536.1 | 1.5 | 11.615 | Unknown gene |
| ltu-miRn118 | AT5G55080.1 | 2.5 | 15.122 | AtRAN4 (Ras-related nuclear protein 4), GTP binding / GTPase/ protein binding |
| ltu-miRn119 | AT2G24300.2 | 2.5 | 23.505 | Calmodulin-binding protein |
| ltu-miRn120 | AT3G45820.1 | 1 | 19.245 | Unknown protein |
| ltu-miRn124 | AT1G66930.1 | 2.5 | 9.562 | Serine/threonine protein kinase family protein |
| ltu-miRn125 | AT2G03667.1 | 2 | 22.07 | Asparagine synthase |
| ltu-miRn128 | AT4G17410.1 | 2.5 | 12.892 | Zinc ion binding |
| ltu-miRn131 | AT1G33710.1 | 3 | 15.3 | RNA-directed DNA polymerase |
| ltu-miRn132 | AT5G05170.1 | 2.5 | 14.979 | CESA3, IXR1, ATCESA3, ATH-B, CEV1 (CONSTITUTIVE EXPRESSION OF VSP 1), cellulose synthase/ transferase, transferring glycosyl groups |
| ltu-miRn134 | AT4G24972.1 | 0.5 | 17.961 | TPD1 (TAPETUM DETERMINANT 1) |
| ltu-miRn136 | AT5G33330.1 | 2.5 | 20.929 | RNase H domain-containing protein |
| ltu-miRn141 | AT1G42470.1 | 1 | 18.322 | Patched family protein |
| ltu-miRn143 | AT5G53440.1 | 1 | 20.268 | Unknown protein |
| ltu-miRn144 | AT3G23270.1 | 1 | 20.289 | RCC1 (regulator of chromosome condensation) family protein |
| ltu-miRn147 | AT1G51620.1 | 1 | 9.414 | Protein kinase family protein |
| ltu-miRn149 | AT2G23945.1 | 1.5 | 18.072 | Chloroplast nucleoid DNA-binding protein-related |
| ltu-miRn150 | AT2G35880.1 | 0.5 | 13.408 | Targeting for Xklp2 |
| ltu-miRn152 | AT2G16570.1 | 2 | 7.514 | ATASE, ATASE1 (GLN PHOSPHORIBOSYL PYROPHOSPHATE AMIDOTRANSFERASE 1), amidophosphoribosyltransferase |
| ltu-miRn153 | AT5G15020.1 | 1.5 | 7.784 | SNL2 (SIN3-LIKE 2) |
| ltu-miRn154 | AT4G24040.1 | 2 | 2.883 | ATTRE1, TRE1 (TREHALASE 1); alpha,alpha-trehalase/ trehalase |
| ltu-miRn156 | AT1G55630.1 | 1.5 | 17.926 | PPR repeat-containing protein |
| ltu-miRn159 | AT5G06560.1 | 1.5 | 11.124 | Unknown protein |
| ltu-miRn163 | AT1G21640.1 | 3 | 2.333 | NADK2, ATNADK2, NAD+ kinase/ calmodulin binding |
| ltu-miRn165 | AT3G46300.1 | 2.5 | 15.923 | Unknown protein |
| ltu-miRn172 | AT5G20170.1 | 1.5 | 15.784 | Unknown protein |
| ltu-miRn173 | ATCG00770.1 | 1.5 | 15.757 | RPS8 (chloroplast 30S ribosomal protein S8) |
| ltu-miRn174 | AT3G21481.1 | 2.5 | 15.079 | Pseudogene of AT4G34870, ROC5 (ROTAMASE CYP 5), peptidyl-prolyl cis-trans isomerase |
| ltu-miRn175 | AT1G02110.1 | 2 | 17.43 | Proline-rich family protein |
| ltu-miRn176 | AT4G03437.1 | 1.5 | 13.269 | Pseudogene of AT4G03480, ankyrin repeat family protein |
| ltu-miRn177 | AT1G59750.3 | 1.5 | 10.477 | ARF1 (AUXIN RESPONSE FACTOR 1), DNA binding / transcription factor |
| ltu-miRn178 | AT2G39230.1 | 1.5 | 16.035 | PPR repeat-containing protein |
| ltu-miRn181 | AT3G17970.1 | 2.5 | 13.503 | AtToc64-III (Arabidopsis thaliana translocon at the outer membrane of chloroplasts 64-III), binding / carbon-nitrogen ligase |
| ltu-miRn182 | AT5G61910.2 | 2 | 19.673 | Development and cell death domain (InterPro:IPR013989), Kelch related (InterPro:IPR013089) |
| ltu-miRn186 | AT1G42350.1 | 1.5 | 11.326 | Transposable element gene |
| ltu-miRn187 | AT5G67630.1 | 2 | 13.261 | DNA helicase, putative |
| ltu-miRn188 | AT2G17050.1 | 2.5 | 15.85 | Disease resistance protein (TIR-NBS-LRR class), putative |
| ltu-miRn189 | AT1G67510.1 | 2 | 10.708 | Leucine-rich repeat family protein |
| ltu-miRn190 | AT3G43546.1 | 1.5 | 20.524 | Transposable element gene |
| ltu-miRn194 | AT4G27220.1 | 3 | 19.024 | Disease resistance protein (NBS-LRR class), putative |
| ltu-miRn196 | AT3G42993.1 | 2.5 | 16.491 | Transposable element gene |
| ltu-miRn197 | AT5G44790.1 | 2 | 16.259 | RAN1 (RESPONSIVE-TO-ANTAGONIST 1) |
| ltu-miRn201 | AT5G58784.1 | 2 | 6.011 | Dehydrodolichyl diphosphate synthase |
| ltu-miRn202 | AT2G29740.1 | 3 | 20.078 | UGT71C2 (UDP-GLUCOSYL TRANSFERASE 71C2) |
| ltu-miRn205 | AT5G61500.1 | 1.5 | 12.283 | ATATG3, ATG3 |
| ltu-miRn207 | AT4G18130.1 | 2 | 18.455 | PHYE (PHYTOCHROME DEFECTIVE E) |
| ltu-miRn208 | AT2G18860.2 | 1 | 16.841 | Syntaxin family protein |
| ltu-miRn209 | AT5G46970.1 | 1 | 18.975 | Invertase/pectin methylesterase inhibitor family protein |
| ltu-miRn213 | AT5G53810.1 | 1.5 | 16.33 | O-methyltransferase, putative |
| ltu-miRn215 | AT1G64260.1 | 1.5 | 22.183 | Zinc finger protein-related |
| ltu-miRn217 | AT5G48120.1 | 1.5 | 17.651 | Binding |
| ltu-miRn221 | AT4G38870.1 | 1.5 | 15.53 | F-box family protein |
| ltu-miRn222 | AT4G39520.1 | 2.5 | 20.584 | GTP-binding protein, putative |
| ltu-miRn225 | AT1G55970.1 | 2 | 15.961 | HAC04, HAG04, HAC4, HAG4, HAC6, histone acetyltransferase |
| ltu-miRn229 | AT2G06540.1 | 2 | 18.168 | Transposable element gene |
| ltu-miRn230 | AT3G18970.1 | 2.5 | 6.063 | PPR repeat-containing protein |
| ltu-miRn231 | AT5G56520.1 | 2 | 21.323 | Unknown protein |
| ltu-miRn232 | AT2G19350.1 | 1.5 | 14.262 | Unknown protein |
| ltu-miRn233 | AT5G51200.1 | 1.5 | 17.531 | Unknown protein |
| ltu-miRn234 | AT3G23810.1 | 2.5 | 19.387 | ATSAHH2, SAHH2 (S-ADENOSYL-L-HOMOCYSTEINE (SAH) HYDROLASE 2), adenosylhomocysteinase/ binding / catalytic |
| ltu-miRn235 | AT5G53810.1 | 1.5 | 16.33 | O-methyltransferase, putative |
| ltu-miRn237 | AT4G23930.2 | 2.5 | 11.271 | Harpin-induced 1 (InterPro:IPR010847), proline-rich family protein |
| ltu-miRn239 | AT1G16640.1 | 1.5 | 13.151 | Transcriptional factor B3 family protein |
| ltu-miRn240 | AT1G30800.1 | 2.5 | 15.727 | Zinc finger (GATA type) family protein |
| ltu-miRn241 | AT3G42791.1 | 1.5 | 16.862 | Transposable element gene |
| ltu-miRn242 | AT5G66620.1 | 1.5 | 14.14 | DAR6 (DA1-RELATED PROTEIN 6); zinc ion binding |
| ltu-miRn243 | AT1G56540.1 | 2.5 | 20.141 | Disease resistance protein (TIR-NBS-LRR class), putative |
| ltu-miRn247 | AT2G44800.1 | 1.5 | 8.429 | Oxidoreductase, 2OG-Fe(II) oxygenase family protein |
| ltu-miRn249 | AT1G18580.1 | 3 | 16.386 | GAUT11 (galacturonosyltransferase 11); polygalacturonate 4-alpha- galacturonosyl -transferase |
| ltu-miRn252 | AT5G38240.1 | 2 | 19.438 | Serine/threonine protein kinase, putative |
| ltu-miRn254 | AT5G48375.1 | 2.5 | 13.641 | TGG3 (THIOGLUCOSIDE GLUCOSIDASE 3), BGLU39; hydrolase, hydrolyzing O-glycosyl compounds / thioglucosidase |
| ltu-miRn255 | AT1G25530.1 | 2.5 | 7.782 | Lysine and histidine specific transporter, putative |
| ltu-miRn257 | AT1G58470.1 | 2.5 | 13.763 | ATRBP1 (ARABIDOPSIS THALIANA RNA-BINDING PROTEIN 1) , RBP1 ; RNA Binding / single-stranded RNA binding |
| ltu-miRn259 | AT1G72590.1 | 1.5 | 11.816 | 3-oxo-5-alpha-steroid 4-dehydrogenase family protein / steroid 5-alpha-reductase family protein |
| ltu-miRn260 | AT3G19025.1 | 2.5 | 10.478 | Pseudogene( similar to ethylene-induced esterase) |
| ltu-miRn261 | AT1G77980.1 | 2 | 15.967 | AGL66 (AGAMOUS-LIKE 66); transcription factor |
| ltu-miRn262 | AT4G21380.1 | 1 | 18.561 | ARK3 (A. THALIANA RECEPTOR KINASE 3); kinase/ transmembrane receptor protein serine/threonine kinase |
| ltu-miRn265 | AT2G04040.1 | 1.5 | 19.695 | ATDTX1, TX1; antiporter/ multidrug efflux pump/ multidrug transporter/ transporter |
| ltu-miRn267 | AT4G32790.1 | 2.5 | 14.889 | Exostosin family protein |
| ltu-miRn269 | AT3G60120.1 | 1.5 | 13.983 | BGLU27 (BETA GLUCOSIDASE 27); catalytic/ cation binding / hydrolase, hydrolyzing O-glycosyl compounds |
| ltu-miRn270 | AT1G68440.1 | 1.5 | 20.563 | Unknown protein |
| ltu-miRn271 | AT4G10530.1 | 2 | 12.384 | Subtilase family protein |

^a^The scoring schema to score the complementarity between small RNA (mainly including miRNA and ta-siRNA) and their target transcript. In this study, we set 2.0 as the threshold of maximum expectation.

^b^ Unpaird energy, maximum energy allowed to unpair the target site.
