# Supplementary material for: Deep Sequencing and Microarray Hybridization Identify Conserved and Species-Specific MicroRNAs during Somatic Embryogenesis in Hybrid Yellow Poplar
Source: PLoS One. 2012 Aug 29;7(8):e43451. doi: 10.1371/journal.pone.0043451 (PMC3430688; doi:10.1371/journal.pone.0043451)
Supplement: Table S6 — The mature miRNA sequences and primers used for RT-PCR and qRT-PCR. (DOC) [file pone.0043451.s007.doc]

**Table S6. The mature miRNA sequences and primers used for RT-PCR and qRT-PCR.**

| **Name** | | **sequence(5` to 3`)** |
| --- | --- | --- |
| miR894 | miRNA sequence | GUUUCACGUCGGGUUCACCA |
|  | Stem-loop RT primer | CTCAACTGGTGTCGTGGAGTCCGGCAATTCAGTTGATGGTGAAC |
|  | Forward primer | ACACTCCAGCTGGGGTTTCACGTC |
| miR165b | miRNA sequence | UCGGACCAGGCUUCAUCCCCC |
|  | Stem-loop RT primer | CTCAACTGGTGTCGTGGAGTCCGGCAATTCAGTTGAGGGGGATG |
|  | Forward primer | ACACTCCAGCTGGGTCGGACCAGG |
| miR166q | miRNA sequence | UCGGACCAGGCUUCAUUCCCC |
|  | Stem-loop RT primer | CTCAACTGGTGTCGTGGAGTCCGGCAATTCAGTTGAGGGGAATG |
|  | Forward primer | ACACTCCAGCTGGGTCGGACCAGG |
| miR319a | miRNA sequence | UUGGACUGAAGGGAGCUCCC |
|  | Stem-loop RT primer | CTCAACTGGTGTCGTGGAGTCCGGCAATTCAGTTGAGGGAGCTC |
|  | Forward primer | ACACTCCAGCTGGGTTGGACTGAA |
| miR162a | miRNA sequence | UCGAUAAACCUCUGCAUCCGG |
|  | Stem-loop RT primer | CTCAACTGGTGTCGTGGAGTCCGGCAATTCAGTTGACCGGATGC |
|  | Forward primer | ACACTCCAGCTGGGTCGATAAACC |
| miR396e | miRNA sequence | CUCAAGAAAGCUGUGGGAAA |
|  | Stem-loop RT primer | CTCAACTGGTGTCGTGGAGTCCGGCAATTCAGTTGATTTCCCAC |
|  | Forward primer | ACACTCCAGCTGGGCTCAAGAAAG |
| miR159d | miRNA sequence | UUUGGAUUGAAGGGAGCUC |
|  | Stem-loop RT primer | CTCAACTGGTGTCGTGGAGTCCGGCAATTCAGTTGAGAGCTCCC |
|  | Forward primer | ACACTCCAGCTGGGTTTGGATTGA |
| miR390d | miRNA sequence | AAGCUCAGGAGGG AUAGCGCC |
|  | Stem-loop RT primer | CTCAACTGGTGTCGTGGAGTCCGGCAATTCAGTTGATATCGCGG |
|  | Forward primer | ACACTCCAGCTGGGAAGCTCAGGA |
| miR482a | miRNA sequence | UCUUGCCGACUCCUCCCAUUCC |
|  | Stem-loop RT primer | CTCAACTGGTGTCGTGGAGTCCGGCAATTCAGTTGAGGAATGGG |
|  | Forward primer | ACACTCCAGCTGGGTCTTGCCGAC |
| miR397b | miRNA sequence | UCAUUGAGUGCAGCGUUGATG |
|  | Stem-loop RT primer | CTCAACTGGTGTCGTGGAGTCCGGCAATTCAGTTGACATCAACG |
|  | Forward primer | ACACTCCAGCTGGGTCATTGAGTG |
| miR2118 | miRNA sequence | UUUCCGAUGCCUCCCAUGCCUA |
|  | Stem-loop RT primer | CTCAACTGGTGTCGTGGAGTCCGGCAATTCAGTTGATAGGCATG |
|  | Forward primer | ACACTCCAGCTGGGTTTCCGATGC |
| ltu-miRn1 | miRNA sequence | AUCUCUGACAGCGGCACGUGGCCC |
|  | Stem-loop RT primer | CTCAACTGGTGTCGTGGAGTCCGGCAATTCAGTTGAGGGCCACG |
|  | Forward primer | ACACTCCAGCTGGGATCTCTGACA |
| ltu-miRn3 | miRNA sequence | UUCCCAAUUCCUCCCAUGCCGU |
|  | Stem-loop RT primer | CTCAACTGGTGTCGTGGAGTCCGGCAATTCAGTTGAACGGCATG |
|  | Forward primer | ACACTCCAGCTGGGTTCCCAATTC |
| ltu-miRn4 | miRNA sequence | UUUCAACACUGAGGUCAUGGG |
|  | Stem-loop RT primer | CTCAACTGGTGTCGTGGAGTCCGGCAATTCAGTTGACCCATGAC |
|  | Forward primer | ACACTCCAGCTGGGTTTCAACACT |
| ltu-miRn5 | miRNA sequence | UUCCCCAAGCCUCCCAUGCCGA |
|  | Stem-loop RT primer | CTCAACTGGTGTCGTGGAGTCCGGCAATTCAGTTGATCGGCATG |
|  | Forward primer | ACACTCCAGCTGGGTTCCCCAAGC |
| ltu-miRn8 | miRNA sequence | UUAGACGACUCUCGGCAAC |
|  | Stem-loop RT primer | CTCAACTGGTGTCGTGGAGTCCGGCAATTCAGTTGAGTTGCCGA |
|  | Forward primer | ACACTCCAGCTGGGTTAGACGACT |
| ltu-miRn86 | miRNA sequence | UUUGGAUCUGCCUCAUUUUUG |
|  | Stem-loop RT primer | CTCAACTGGTGTCGTGGAGTCCGGCAATTCAGTTGACAAAAATG |
|  | Forward primer | ACACTCCAGCTGGGTTTGGATCTG |
| Universal | Reverse primer | AACTGGTGTCGTGGAG |
| LtuLAC-like | Forward primer | TACGAATCATCAACGCTGCACTC |
|  | Reverse primer | GGCCTGGGCCTAAGACAAC |
| 5S rRNA | Forward primer | ACACCAATCCATCCCGAACT |
|  | Reverse primer | TCCTGGCGTCGAGCTATTT |
| 18S rRNA | Forward primer | GCCCGTCGCTCTGATGAT |
|  | Reverse primer | CTGCCTTCCTTGGATGTGGT |
